# Analysis of an improved *Cyanophora paradoxa* genome assembly

**DOI:** 10.1093/dnares/dsz009

**Published:** 2019-05-16

**Authors:** Dana C Price, Ursula W Goodenough, Robyn Roth, Jae-Hyeok Lee, Thamali Kariyawasam, Marek Mutwil, Camilla Ferrari, Fabio Facchinelli, Steven G Ball, Ugo Cenci, Cheong Xin Chan, Nicole E Wagner, Hwan Su Yoon, Andreas P M Weber, Debashish Bhattacharya

**Affiliations:** 1 Department of Plant Biology, Rutgers, The State University of New Jersey, New Brunswick, NJ, USA; 2 Department of Biology, Washington University, St. Louis, MO, USA; 3 Washington University Center for Cellular Imaging, Washington University School of Medicine, St. Louis, MO, USA; 4 Department of Botany, University of British Columbia, Vancouver, BC, Canada; 5 Department of Molecular Physiology, Max Planck Institute of Molecular Plant Physiology, Potsdam, Germany; 6 School of Biological Sciences, Nanyang Technological University, Singapore; 7 Institute for Plant Biochemistry, Cluster of Excellence on Plant Sciences (CEPLAS), Heinrich-Heine-University, D-40225 Düsseldorf, Germany; 8 Unité de Glycobiologie Structurale et Fonctionnelle, UMR 8576 CNRS-USTL, Université des Sciences et Technologies de Lille, Villeneuve d’Ascq Cedex, France; 9 Institute for Molecular Bioscience and School of Chemistry and Molecular Biosciences, The University of Queensland, Brisbane, QLD, Australia; 10 Department of Biochemistry and Microbiology, Rutgers, Rutgers University, New Brunswick, NJ, USA; 11 Department of Biological Sciences, Sungkyunkwan University, Suwon, Korea

**Keywords:** *Cyanophora paradoxa*, Archaeplastida, phylogenomics, tree of eukaryotes, ultrastructure

## Abstract

Glaucophyta are members of the Archaeplastida, the founding group of photosynthetic eukaryotes that also includes red algae (Rhodophyta), green algae, and plants (Viridiplantae). Here we present a high-quality assembly, built using long-read sequences, of the ca. 100 Mb nuclear genome of the model glaucophyte *Cyanophora paradoxa*. We also conducted a quick-freeze deep-etch electron microscopy (QFDEEM) analysis of *C. paradoxa* cells to investigate glaucophyte morphology in comparison to other organisms. Using the genome data, we generated a resolved 115-taxon eukaryotic tree of life that includes a well-supported, monophyletic Archaeplastida. Analysis of muroplast peptidoglycan (PG) ultrastructure using QFDEEM shows that PG is most dense at the cleavage-furrow. Analysis of the chlamydial contribution to glaucophytes and other Archaeplastida shows that these foreign sequences likely played a key role in anaerobic glycolysis in primordial algae to alleviate ATP starvation under night-time hypoxia. The robust genome assembly of *C. paradoxa* significantly advances knowledge about this model species and provides a reference for exploring the panoply of traits associated with the anciently diverged glaucophyte lineage.

## 1. Introduction

**Figure 1:**
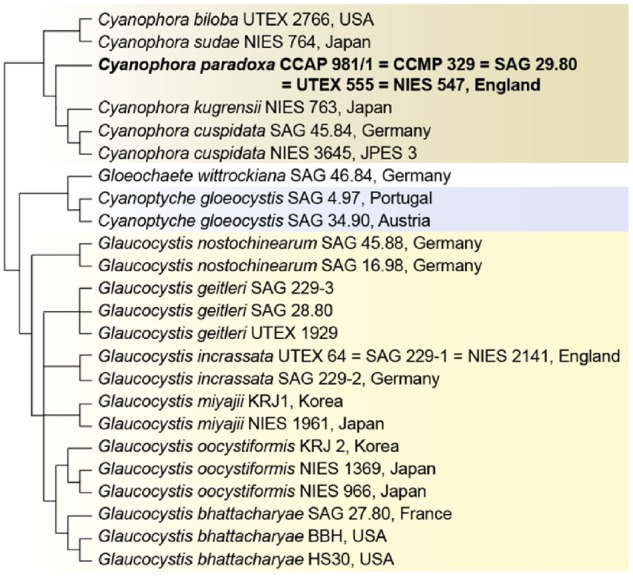
The phylogeny of Glaucophyta. This tree was built using a multigene dataset comprising plastid-encoded PsbA, PsaB, and 16S rRNA, mitochondrial COX1 and COB, and nuclear-encoded ITS regions and partial sequences of SSU and LSU rRNA (modified from Price *et al.*^9^).

**Figure 2:**
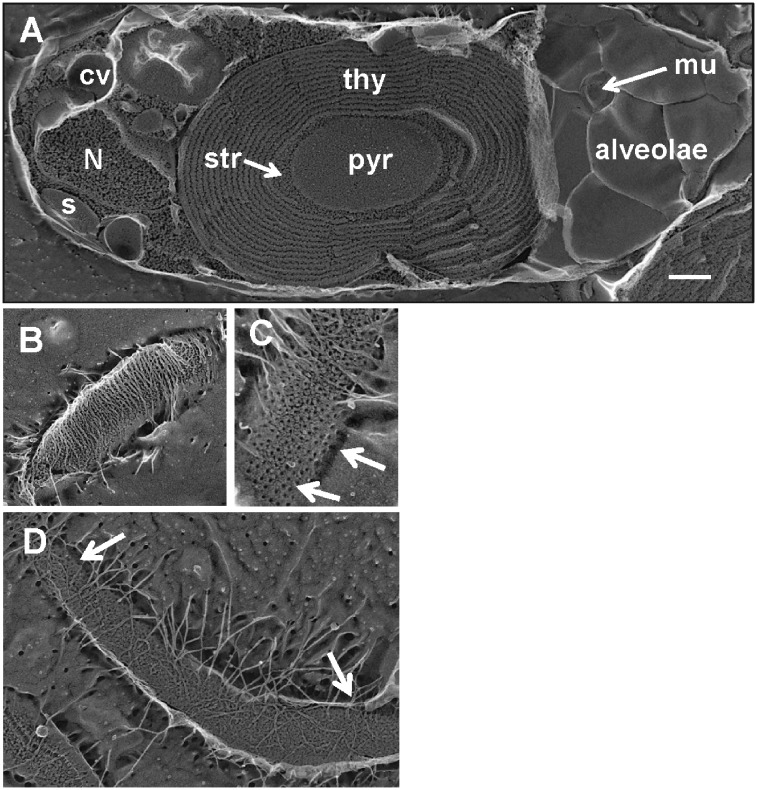
Quick-freeze deep-etch electron microscopy (QFDEEM) images of the biflagellate *Cyanophora paradoxa* cell. (A) The fracture plane passes through the interior of alveolar membranes at right, exposing a mucocyst (mu) poised for secretion, then cross-fractures the plastid with its central pyrenoid (pyr) surrounded by stroma (str) and thylakoids (thy). The nucleus (N), contractile vacuole (cv), and starch (s) are at the anterior end of the cell. The scale bar is 500 nm. (B) Flattened mastigonemes (flagellar hairs) densely decorate one flagellar surface. (C, D) Long, narrow mastigonemes more sparsely decorate the other flagellar surface, which displays donut-shaped units (arrows), as contrasted with the knobbly surface of the plasma membrane.

The glaucophyte algae (Glaucophyta^1^) are a small group of unicellular and colonial taxa with four described genera and about 15 species (Fig. 1). These taxa represent one branch of the Archaeplastida [the others are the red (Rhodophyta) and green algae and plants (Viridiplantae)]^2^ whose common ancestor putatively captured and retained a cyanobacterial endosymbiont ca. 1.6 billion years ago through primary endosymbiosis.^3–6^ The green algae in this ‘supergroup’ gave rise to plants, and the plastids of red and green algae were spread via serial endosymbioses to a myriad of other important primary producers including diatoms, haptophytes, dinoflagellates, and euglenids.^7^ What makes glaucophytes of particular interest is that they uniquely retain a suite of plastid traits associated with the ancestral cyanobacterial endosymbiont, such as peptidoglycan (PG) and phycobilisomes, and lack chlorophyll-*b*.^8^^,^^9^ This lineage also harbours the primordial, bacterial (putatively chlamydial) derived UhpC-type hexose-phosphate transporter to translocate fixed carbon from the plastid to the host cytosol. This system was replaced in the red and green lineages by a complex gene family derived from existing eukaryote nucleotide sugar transporters that diversified into plastid phosphate-translocators.^10^ In addition, the model glaucophyte *Cyanophora paradoxa* (Fig. 2) contains an apparently primitive RNA interference pathway.^11^ Hence glaucophytes provide valuable insights into the presumed putative ancestral state of the Archaeplastida host and its photosynthetic organelle.

Analysis of individual proteins predicted from a prior draft genome assembly of *C. paradoxa* CCMP329 (Pringsheim strain)^10^ not only provided evidence of Archaeplastida monophyly based on single-gene phylogenetic analyses, but also revealed the complex biology of this lineage. For instance, Glaucophyta was found to contain a sophisticated plastid protein import machinery (e.g. the protein translocation channel Toc75; Supplementary Fig. S1) and pathways for starch biosynthesis and fermentation that are shared with other Archaeplastida. These derived traits belied the presumed ‘living fossil’ moniker of this lineage.^10^ Although valuable for assessing the gene inventory, the initial assembly was fragmented due to the primary use of Illumina short-read sequencing technology that was unable to deal adequately with the high average genome GC-content (67.6%) and intron-rich genes. Here we report a significantly improved genome assembly of the same *C. paradoxa* strain derived from PacBio long-read sequencing, in concert with extensive Illumina data for sequence correction. RNA-Seq data derived from cultures that span the light–dark transition in *C. paradoxa* and recently available transcriptome data from three other glaucophytes provide novel information on genome content, allowing us to comprehensively assess the evolutionary trajectory of glaucophytes and to test Archaeplastida monophyly in an expanded multigene tree of life. Furthermore, we provide a detailed analysis of *C. paradoxa* cell ultrastructure to assess existing hypotheses about the morphological evolution of this lineage. These wide-ranging results paint a fascinating picture of glaucophyte evolution and identify features that both unite and distinguish *C. paradoxa* from its Archaeplastida sisters.

## 2. Materials and methods

### 2.1. Genome sequencing

Approximately 100 ml of a log-phase cell culture pellet was ground in liquid nitrogen, re-suspended in 10 ml G2 lysis buffer (Qiagen, Venlo, The Netherlands), and incubated at 37°C for 2 h with gentle agitation. RNase A was added to a final concentration of 20 μg/ml and incubated for 30 min at 37°C prior to addition of Proteinase K (Qiagen) to 0.8 mg/ml and incubation for 2 h at 50°C with gentle agitation. The lysate was centrifuged for 20 min at 12,000–15,000 g to remove insoluble debris, transferred to an equilibrated Qiagen Genomic-tip 20 column, and washed four times with 1 ml of QC buffer (Qiagen) via gravity. The DNA was eluted with 0.8 ml QF buffer (Qiagen) via gravity, precipitated with 0.7 volumes of RT isopropanol and centrifuged for 20 min at 15,000 g. The supernatant was discarded, the pellet was washed with ice-cold 70% ethanol, allowed to air-dry, and re-suspended in 50 μl of TE. The DNA was sent to the Max Planck Institute genome sequencing centre for library construction (Cologne, Germany) and six SMRT cells were sequenced on an RSII instrument using P6-C4 chemistry, producing over 7.46 Gb of long-read sequence data with an average insert size of ca. 15 kb. The raw reads were trimmed, self-corrected and assembled using the Canu pipeline v1.0^12^ with the following parameters: genomeSize = 110m, minOverlapLength = 1,000, utgGraphErrorRate = 0.020, utgRepeatErrorRate = 0.020. Two successive rounds of genome polishing were performed with Quiver (https://github.com/PacificBiosciences/GenomicConsensus (date last accessed 12 April 2019)) and a final correction was performed by mapping the Illumina genomic DNA libraries to the assembly using the CLC Genomics Workbench (95% similarity over 95% of the read length) and calling bases on a majority-rule basis with areas of zero coverage filled from the PacBio reference. Based on a cursory BLASTx analysis against bacterial genomes available at NCBI, we removed 36 contigs representing partial bacterial contaminant fragments to arrive at a final assembly spanning 712 contigs and totalling 99.94 Mb with a contig N50 = 214 kb.

### 2.2. Illumina RNA-Seq

Approximately 50 ml of log-phase *C. paradoxa* culture was centrifuged for 2 min and the cell pellet was ground in liquid nitrogen prior to extraction using TRIzol (Thermo Fisher Scientific) per the manufacturer’s protocol. Approximately 100 ng of total RNA was used to prepare an Illumina RNA-Seq library with the RNA Sample prep kit v2 (Illumina, Inc.) and sequenced on a MiSeq instrument in 2 ×250 bp paired-end mode using a 500-cycle MiSeq reagent kit V2 (Illumina, Inc.). These reads were quality and adapter trimmed using the CLC Genomics Workbench, assembled to transcripts using Trinity^13^ in both a *de novo* and genome-guided manner, and clustered at 95% sequence similarity using CD-HIT.^14^ Evidence of long overlapping ORFs on these transcripts was found by translating with EMBOSS.^15^ We produced over 43.6 million RNA-Seq reads totalling 10.9 Gb of data. These reads assembled into 238,820 EST contigs. An additional 51 million stranded RNA-Seq reads were generated using 1.3 μg of extracted RNA with the TruSeq Stranded mRNA sample prep kit and sequenced in 2×75 bp paired-end mode.

### 2.3. Illumina genome data

Approximately 50 ml of log-phase *C. paradoxa* culture was centrifuged for 2 min and the cell pellet was extracted using the Qiagen DNeasy Plant DNA kit. Two aliquots of ca. ∼200 ng of genomic DNA were each sheared using a Covaris ultrasonicator (Covaris, Inc., Woburn, MA) to 500 and 800 bp, respectively, and used to construct two individual sequencing libraries using the TruSeq Nano DNA Sample prep kit (Illumina, Inc.) that were then sequenced on a MiSeq instrument in 2×300 bp paired-end mode using a 600-cycle MiSeq reagent kit v3 (Illumina, Inc.). Two sequencing runs were performed: one with the 500 and 800 bp inserts multiplexed, and another with the 500 bp insert alone. In total we produced 109 million reads totalling 33.59 Gb of *C. paradoxa* genomic DNA. Reads were quality and adapter trimmed using the CLC Genomics Workbench (Qiagen).

### 2.4. Analysis of repeat content

We compared the repeat content of the *C. paradoxa* genome against those in red and green algal genomes. To minimize potential biases of misassembled repetitive regions, we focussed only on available genome assemblies that comprise highly contiguous sequences or at chromosomal resolution: three red algae (*Cyanidioschyzon merolae*^16^ [NCBI BioProject PRJNA10792], *Gracilariopsis chorda*^17^ [PRJNA361418], and *Porphyra umbilicalis*^18^ v1.5 [PRJNA234409]), and three green algae (the prasinophytes, *Micromonas commoda*^19^ RCC299 [PRJNA15676] and *Ostreococcus lucimarinus*^20^ CCE9901 [PRJNA13044], and *Chlamydomonas reinhardtii*^21^ v5.5 [PRJNA12260]). For each genome assembly, a *de novo* repeat library was first derived using RepeatModeler v1.0.11 (http://repeatmasker.org/RepeatModeler (date last accessed 12 April 2019)) at default setting. All repeats were then identified using RepeatMasker v4.0.6 (http://repeatmasker.org (date last accessed 12 April 2019)) based on evidence in the customized repeat library (i.e. the RepeatMasker database plus the *de novo* repeats identified above). The repeat landscape for each genome was generated using the utility script *calcDivergenceFromAlign.pl* from RepeatMasker.

### 2.5. Gene prediction

The RNA-Seq reads were mapped to genome contigs using STAR^22^ and the resulting .bam file was used in the BRAKER1 pipeline^23^ to ultimately train Augustus^24^ in predicting genes across the *C. paradoxa* genome. Augustus predicted 25,831 genes encoded on the *C. paradoxa* contigs. Each translated protein sequence was used in a BLASTp query against the NCBI ‘nr’ database (*E*-val =1×10^−5^) to assign a provisional annotation.

### 2.6. Single nucleotide polymorphism detection

The 109 million genomic DNA reads were mapped to the PacBio scaffolds using the CLC Genomics Workbench (95% similarity fraction over 95% of read length) and SNPs were called using the CLC Genomics Workbench variant caller (non-specific matches ignored, min. coverage = 10x, min. count = 3, min. central quality = 20).

### 2.7. Phylogenomics of individual predicted proteins

Each predicted *C. paradoxa* protein was used in a BLASTp query against an in-house database composed of NCBI RefSeq v. 73 proteins with the addition of available algal and protist genome and transcriptome data from dbEST, TBestDB, the JGI Genome Portal (https://genome.jgi.doe.gov (date last accessed 12 April 2019)) and the Microbial Eukaryote Transcriptome Sequencing Project (MMETSP).^25^ This database was partitioned into four subsets based on taxonomic provenance: Bacteria, Opisthokonta, remaining non-bacterial or opisthokont taxa, and the MMETSP database. Each subset was searched against independently (BLASTp, *E *≤* *10^−10^) using the *C. paradoxa* proteins and the top 2,000 hits from each search were saved, combined, and sorted by bitscore. The sorted list was parsed such that a taxonomically broad selection of top hits was retained, and the associated proteins were aligned together with the query sequence using MAFFT v7.2.^26^ Maximum-likelihood phylogenetic trees were constructed using IQTREE v1.3 (81) after automatic model selection with nodal support tested via 2,000 ultrafast phylogenetic bootstraps.^27^

### 2.8. Differential gene-expression analysis

Three biological replicates of *C. paradoxa* cells grown in a 16: 8 L/D cycle were each harvested and flash-frozen in liquid nitrogen prior to RNA extraction at the following 6 timepoints: 1 h after lights-on (ALO), 8 h ALO, 14 h ALO, 1 h after dark (AD), 5 h AD, 7 h AD. The TruSeq RNA sample prep kit (Illumina, Inc.) was used to prepare Illumina RNAseq libraries for each (18 total libraries) prior to sequencing on an Illumina HiSeq using 50 bp single-end sequencing reagents. Approximately 20–24 million reads were generated for each library that were then mapped to the genome contigs using STAR.^22^ The resulting .bam files were parsed to generate per-gene counts with HTSeq 0.9.1^28^ that were combined in a matrix and read with the R Bioconductor DEseq2 package.^29^ Read counts were normalized, and any gene that: (i) exhibited a log2 fold change of (+/−) 1.5 or greater between the first time point (1ALO) and any other time point, and (ii) had a BLASTp hit to the NCBI nr database, was retained as differentially expressed (DE). A variance stabilizing transformation was applied, and the set of DE genes was then clustered using an agglomerative hierarchical method available via the ‘agnes’ function. The variance-stabilized data pertaining to cluster members were then plotted and inspected; clusters were manually split, joined and outlying members were removed.

To determine whether any clusters exhibited patterns of functional enrichment, the *C. paradoxa* predicted proteome was annotated using Blast2GO,^30^ and member genes of each cluster were used in a Fisher’s exact test, as performed in Blast2GO, with the reference set consisting of all 12,652 genes with BLAST hits in the genome. Gene Ontologies with an uncorrected *P*-value of <0.05 were retained. In addition, genes were annotated via the KEGG Automatic Annotation Server (KAAS, https://www.genome.jp/kegg/kaas/^31^ (date last accessed 12 April 2019)) and grouped according to KEGG pathway assignment. Plots were created to illustrate the overall transcriptional response of all members in each pathway; those with a clear up- or down-ward trend comprising the majority of assigned genes were retained for discussion. In all, we identified 4,297 differentially expressed genes in the *C. paradoxa* light/dark treatment. These genes clustered into 40 predominant expression patterns. The cell cycle genes were identified via BLASTp using curated homologues from other algae, and further qualified using the NCBI ‘nr’ databases.

### 2.9. Eukaryote tree of life

To place *C. paradoxa* in a broader eukaryote tree of life and test Archaeplastida monophyly, we adapted and expanded the protocol of Price and Bhattacharya^32^ to derive *de novo* ortholog groups (OGs) and construct a 3,000 OG dataset from 115 publicly available eukaryote proteomes. Briefly, EST and/or predicted proteome data were retrieved for each species (see Supplementary Table S1) and OrthoFinder^33^ was used to construct ortholog groups (OGs) from the total data. We parsed each group (or putative gene family) and retained those that had low levels of paralogy (>80% of genes in the group were single-copy [i.e. one protein per taxon]) and contained ≥4 phyla and ≥10 species. Taxa with multi-copy representative proteins were removed from these groups, and the protein sequences corresponding to each individual group were aligned with MAFFT v. 7.3.^26^ These alignments were then used to construct a maximum-likelihood phylogeny using IQ-TREE^34^ via a partitioned analysis in which each OG alignment represented a single partition with unlinked models of evolution chosen by IQTREE. A consensus tree was generated from the combined bootstrap set, and node support was determined by 2,000 rapid (UFBoot) bootstraps.

## 3. Results and discussion

### 3.1. Genome data and repeat content

We sequenced six PacBio RSII SMRT cells of *C. paradoxa* genomic DNA, producing over 7.46 Gb of long-read sequence data (see Supplementary Methods). A BLASTx analysis of the PacBio assembly using the NCBI bacterial proteome database identified 36 contigs containing partial bacterial contaminant fragments. These were removed yielding the final assembly of 712 contigs totalling 99.94 Mb (N50 = 214 kb). Inspection of various *k*-mer spectra under the expectation of haploidy or diploidy (http://kmergenie.bx.psu.edu (date last accessed 12 April 2019)) using high-quality Illumina reads provided evidence that *C. paradoxa* is more likely to be a haploid than a highly homogenized diploid genome (Supplementary Fig. S2). In support of this hypothesis, a SNP analysis using the PacBio assembly and Illumina short-read data identified 27,675 variants with at least 10x coverage (using 95% sequence identity over 95% of the trimmed read length). Of these, only 5,016 (or 18%) occurred at a frequency between 30–70%, suggesting that the major class of SNPs, expected at 40–60% for a diploid, was not recovered. Applying an *ab initio* approach for gene prediction guided by transcriptome data, we predicted 25,831 protein-coding genes in the *C. paradoxa* genome (excluding bidirectional transcripts, see below). Genes were densely packed, spanning 60.3% of the genome with a mean intergenic distance of only 770 bp (likely an over-estimation given that we did not predict UTRs), often oriented end-to-end with many short introns (mean = 9.2 introns/gene; mean length = 86 bp). Such complex gene structures are problematic for HMM-based gene predictors therefore the models presented here will require additional manual curation. BUSCO^35^ was used to assess completeness of the predicted proteome and identified 282 of the 303 (93%; 90% complete, 3% fragmented) of the core proteins as being present in the eukaryote (odb9) dataset, suggesting that the assembly was nearly complete. This represents a major improvement over the previous *C. paradoxa* predicted proteome^10^ that, although found to contain 273 (90%) of core odb9 proteins, was highly fragmented (58% complete, 32% fragmented).

To assess the difference in assembly size between the previous (70 Mb) and the current assembly, we aligned the two assemblies using MUMmer 4.0b2 (https://github.com/mummer4/mummer (date last accessed 12 April 2019)) and find that 65.2 Mb of the assembly maps one-to-one between the two genomes, with 87,101 duplicate features annotated (type ‘DUP’) in the new assembly that sum to 34.7 Mb. The one-to-one and duplicate genome feature lengths sum to 99.9 Mb, or the length of the new assembly, and thus the previous assembly size was likely an artefact of repetitive, paralogous and/or multi-copy DNA content that was resolved here with the use of long-read PacBio sequencing. Short-read mapping of 105 million Illumina reads to the new assembly indicates that 91.6% of reads map successfully, with 17.9 million (18.5%) having more than one best placement. A BLASTP analysis (*e*-val =1×10^−10^) indicates that 22,678 of 25,831 (87.8%) predicted proteins from the new assembly have hits to the previous proteome (Supplementary Tables S2 and S5), whereas 27,066 of 32,167 (84.1%) predicted proteins from the old assembly have hits to the current proteome (Supplementary Table S3). Of the 5,101 proteins from the previous assembly with no hits, 2,355 have TBLASTN hits (*e*-val =1×10^−10^) to the new genome, however only 194 have a BLASTP hit to the NCBI nr database (Supplementary Table S4).

We analysed repeat content in the *C. paradoxa* genome and found that 29.4% is comprised of repetitive elements and 11.5% is unknown repeats (i.e. novel repeats). The repeat landscape of *C. paradoxa* (Supplementary Fig. S3) indicates little or no expansion of repetitive elements. Only a small proportion of these sequences are long terminal repeats (LTRs: 1.77% of genome) and long interspersed nuclear elements (LINEs; 2.47% of genome). This is in stark contrast to the prominence of LTRs in red algal genomes (e.g. 35.6% in *Gracilariopsis chorda*) and the greater proportion of LINEs in the genome of the green alga *C. reinhardtii* (5.6%; Supplementary Fig. S3). These results indicate the expansion of LTRs in red algae and LINEs in chlorophytes after the split from Glaucophyta (e.g. *Micromonas commoda*, *Ostreococcus lucimarinus*; Supplementary Fig. S3). The LTRs in the *Cyanidioschyzon merolae* genome^17^ show the highest divergence (Supplementary Fig. S3). In Rhodophyta, the gene inventory has remained relatively small (ca. 5–13 K genes) over >1 billion years of evolution, and the growth of genome size in multicellular red seaweeds is largely explained by transposable element (TE) expansion.^17^ In land plants, TE activity has played a major role in genome size growth but gene families have significantly expanded when compared with green algal groups.^17^ Within this context of Archaeplastida evolution, *C. paradoxa* occupies a unique position for a unicellular lineage by encoding a relatively rich gene inventory (e.g. compared with 16,709 genes in *C. reinhardtii*; https://genome.jgi.doe.gov/Chlre4/Chlre4.home.html (date last accessed 12 April 2019))^21^ but not having undergone large-scale expansion of repetitive elements, as in many plants.

**Figure 3:**
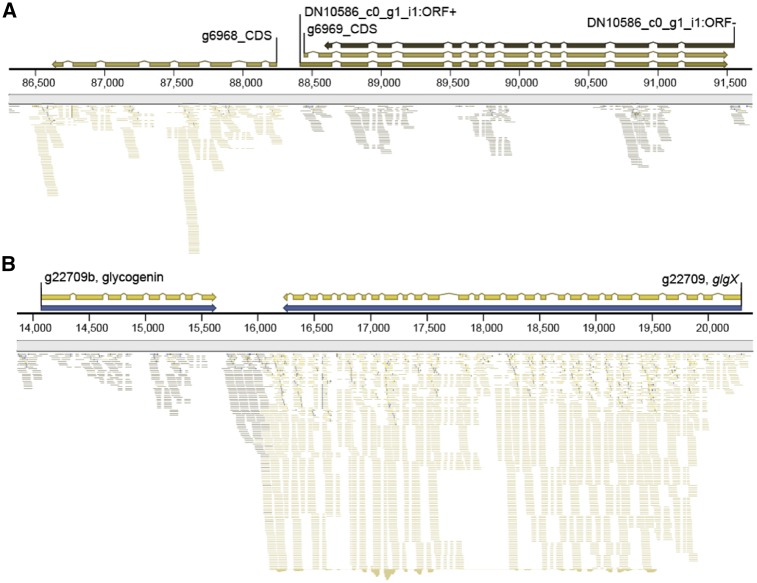
Mapping RNA-Seq reads to a region of the *Cyanophora paradoxa* nuclear genome. (A) Gene structure of a ca. 3,050 bp sequence (DN10586_c0_g1_i1: ORF+) containing 13 introns that encodes overlapping ORFs spanning a gene structure predicted by Augustus. However, only the DN10586_c0_g1_i1: ORF+ has RNA-seq support using stranded transcriptome data. (B) Genomic region showing a chlamydia-derived glycogen debranching enzyme containing 27 introns that shares a predicted 3′ UTR with a eukaryote-derived gene (g22709b) encoding a complete eukaryotic glycogenin (GT8) domain involved in glycogen biosynthesis. The stranded RNA-Seq data are mapped to both genes, showing the region of overlap. Reads in dark grey are transcribed from the plus strand, whereas those in olive or light grey are transcribed from the minus strand.

Interestingly, we find that translation of our assembled *de novo* transcriptome dataset revealed 3,510 non-redundant (excluding alternate isoforms) transcripts (see Supplementary Table S6) that encode bi-directional and partially or completely overlapping open reading frames (ORFs) of at least 300 amino acids (900 nucleotides) in length. One such example (shown in Fig. 3A), a ca. 3,050 bp sequence (DN10586_c0_g1_i1: ORF+) on contig (tig)00001085 contains 13 introns and encodes two bidirectional and overlapping ORFs spanning a gene structure predicted by Augustus. In the forward frame (in relation to the predicted structure), the encoded 718-amino acid protein is a putative bacterial-derived ABC transporter with a local BLASTp top hit spanning the entire length of the query to *Pontibacter chinhatensis* (WP_119433509; 626 AA; *E *=* *0.0; Supplementary Fig. S4). The reverse frame encodes a 715-amino acid protein for which the N-terminal 395 amino acids align via BLASTp to *Oscillibacter* sp. (CDC69327.1; 453 AA; *E *=* *2×10^−22^) annotated as a 30S ribosomal protein S5. To test whether these ORFs may represent bidirectionally transcribed genes, we mapped stranded RNA-Seq data to these gene structures (e.g. Fig. 3A), however we found no evidence of their transcription. We consider it extremely unlikely that nucleotide CDS sequences 1–3 kb in size would by chance encode a conserved (i.e. to known NCBI homologues) protein translation (free of stop codons) in the reverse frame after horizontal gene transfer (HGT) to the *C. paradoxa* nuclear genome and subsequent accrual of 13 spliceosomal introns. Additional work is required to determine the potential biological implication of these findings.

Many genes however show evidence of overlapping UTRs. The chlamydia-derived glycogen debranching enzyme (*glgX* [gene tig0021589_g22709]; Supplementary Fig. S5) for example, contains 27 introns and shares a predicted 3′ UTR with a eukaryote-derived gene (g22709b) encoding a putative glycogenin (GT8) domain that is involved in glycogen biosynthesis (Fig. 3B and Supplementary Fig. S6). This result provides evidence of a bacteria-derived gene that shares a 3′ UTR with a host (eukaryotic) gene, both of which are involved in glycogen metabolism. Bidirectional transcription is often associated with gene regulation via short interfering RNAs [siRNAs, as reported in *C. paradoxa*^11^] or long-non-coding RNAs that are complementary to mRNAs and involved in transcriptional gene silencing.^36–38^ Here, we report that *C. paradoxa* in at least one case co-localizes genes with a related function. The finding of the glycogen gene-pair suggests that other bidirectional and co-expressed genes involved in related functions may be uncovered. If found, this will provide a parallel case to the trebouxiophyte green alga *Picochlorum* SE3, in which gene co-localization may allow a rapid response to variable environmental conditions.^39^^,^^40^

### 3.2. Transcriptomic response to light

We quantified broad patterns of *C. paradoxa* gene expression in response to light by conducting a time-course differential gene-expression analysis using RNA-Seq data collected at six time points spanning a 16: 8 light/dark cycle (see Supplementary Text and Figs S7 and S8). Here, we focussed on genes involved in the cell cycle, recognizing that the circadian clock likely controls some of these functions. Expression of the S- and M-phase specific transcripts encoding PCNA and CycB, respectively^41^ (20) peaked at 8 and 14 h after light exposure (Supplementary Fig. S9), indicating that the cell cycle was likely synchronized in the studied culture. This allowed us to further examine expression patterns of nuclear-encoded cellular and plastid-division-related genes to assess whether coordinated transcriptional regulation occurs during cell cycle progression. Plastid division proceeds via recruitment and assembly of FtsZ filaments by ARC6^42^ prior to constriction of the macromolecular complex. Both *ftsZ* and *arc6* transcripts peak during S-phase within 1 h after exposure to light and decline steadily thereafter, indicating formation of the plastid-dividing (PD) ring on the stromal side. Although Miyagishima *et al.*^41^ reported constant expression of *ftsZ* throughout the cell cycle in cultures synchronized under continuous light using aphidicolin, we find here that significant up-regulation of the transcript occurs in early S-phase. Placement of the PD ring at the future septum is guided by the S-phase specific Min system^43^ for which *minD* and *minE* are present in *C. paradoxa* and peak ∼8 h after light exposure. An outer PD ring has not been observed in *C. paradoxa*,^44^ and eukaryotic host-derived genes for plastid division (e.g. *drp5B*) are absent from the genome, indicating that an alternative mechanism may be responsible for outer envelope division. Although a gene encoding a dynamin-related protein (DRP) that potentially binds the cytosolic plastid division complex was predicted in our assembly, sequence similarity indicates that this is a member of the *drp5A* family and thus linked only to cytokinesis.^45^ PG ingrowth at the division site follows,^46^ and we find that genes encoding proteins involved in PG biosynthesis (*pbp1a*, *murA*, *murB*, *ftsI*; and see below) are up-regulated and peak at ∼8 h after exposure to light, presumably after localization by the Min system. Onset of PD-ring constriction and regulation of the G2/M cycle transition is supported by up-regulation of the mitotic cyclin genes *CycA*, *CycB* and cyclin-dependent kinase B (*CDKB*)^47^ that reach maximum expression levels ∼14 h after exposure to light and begin to drop rapidly 1 h after onset of dark as M-phase progresses. Together, these results indicate transcriptional regulation of nuclear-encoded plastid division genes via the cell cycle as has previously been suggested for Glaucophyta and other algae.^41^ Whether the cell cycle itself in *C. paradoxa* is regulated by a circadian clock mechanism remains unclear, because the main control loops have yet to be established. In addition, an undescribed retrograde signalling pathway likely exists in algae that arrest the cell cycle prior to anaphase until constriction of the plastid division site commences.^48^ Time-course expression data such as those generated in this study will be particularly useful for addressing this issue.

Light exposure also resulted in an overall up-regulation of genes shared between pathways involved in genetic information processing, including those responsible for DNA replication/damage repair (DNA replication, base excision repair, nucleotide excision repair, mismatch repair and homologous recombination) and the cell cycle progression (cell cycle and meiosis). In addition, we found degradation of amino acids (valine, leucine and isoleucine degradation, and lysine degradation) and biosynthesis of *N*- and *O*-linked glycans (*N*-glycan biosynthesis, various types of *N*-glycan biosynthesis, other types of O-glycan biosynthesis) to be up-regulated along with constituents of the endocytosis and butanoate metabolism pathways (Supplementary Fig. S7). In contrast, significant down-regulation was found for genes (Supplementary Fig. S8) assigned to pathways involved in translation and protein trafficking (ribosome [ribosomal proteins], ribosome biogenesis in eukaryotes, aminoacyl-tRNA biosynthesis, protein export) along with isoprenoid biosynthesis (terpenoid backbone biosynthesis, carotenoid biosynthesis), cofactor metabolism (riboflavin metabolism, vitamin B6 metabolism, folate biosynthesis and porphyrin and chlorophyll metabolism), and amino acid metabolism (phenylalanine, tyrosine and tryptophan biosynthesis).

### 3.3. Eukaryote tree of life

**Figure 4:**
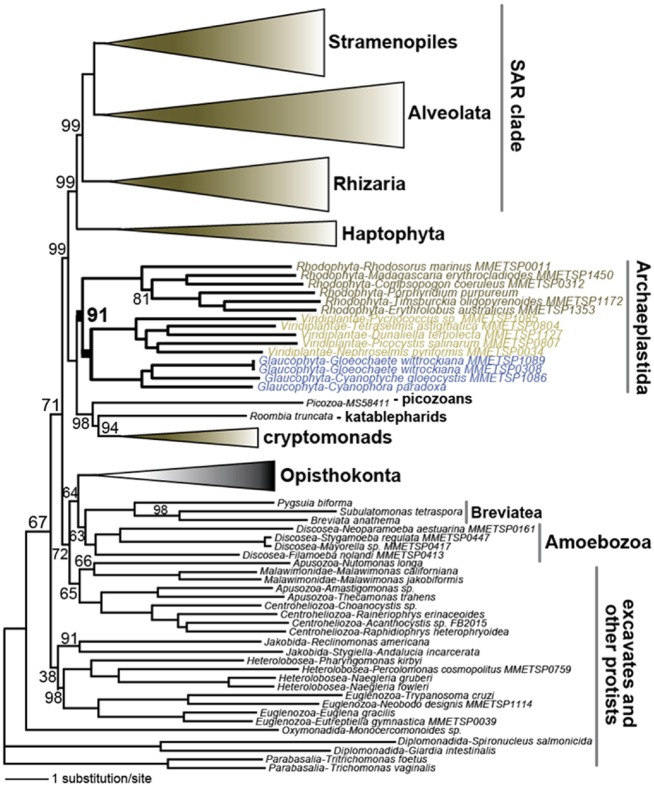
Phylogeny showing the position of *Cyanophora paradoxa* and other glaucophytes in the eukaryotic tree of life. This tree was constructed using a partitioned 3,000 OG dataset from 115 publicly available eukaryote proteomes, as described in the text and reference.^32^ All nodes have 100% bootstrap support unless shown otherwise. The full tree is presented in Supplementary Fig. S10.

To place *C. paradoxa* in a broader tree of life and test for shared ancestry among multiple microbial eukaryote groups, we adapted and expanded the protocol of Price and Bhattacharya^32^ to derive *de novo* ortholog groups (OGs) and constructed a partitioned 3,000 OG dataset from 115 publicly available eukaryote proteomes (Supplementary Table S1). The consensus maximum-likelihood tree (Fig. 4 and Supplementary Fig. S10) is consistent with the monophyly of most major eukaryote phyla as has been previously published (see below). This tree also provides strong support for, and therefore confirms a single origin of the Archaeplastida, whose common ancestor hosted the cyanobacterial primary endosymbiosis that gave rise to the plastid in eukaryotes.^10^^,^^49^^,^^50^ This result is often found when using multigene datasets from the nuclear^51^^,^^52^ and mitochondrial compartments,^53^ but Archaeplastida non-monophyly has also been reported in several cases^52^ using the CAT + GTR + Γ evolutionary model; figure S2 in Burki *et al.*^54^

In support of the monophyly hypothesis, analysis of single genes from the *C. paradoxa* genome by Price *et al.*^10^ showed that of 4,445 maximum-likelihood trees analysed, over 60% supported a sister group relationship between glaucophytes and red and/or green algae plus plants at a bootstrap support ≥90%. In addition to the monophyly of the glaucophyte and green lineages based on plastid-encoded genes, Lee *et al.*^55^ also identified 23 OGs in the nuclear genomes of both lineages that were transferred to the nucleus (i.e. totalling 93 gene families) from the genome of the ancestor of the Glaucophyta + Viridiplantae plastid. This shared history of endosymbiotic gene transfer (EGT) supports the monophyly of these two lineages within Archaeplastida. In comparison, only four such OGs are common to all three lineages, and only one primordial OG is common to the greens and rhodophytes.

**Figure 5:**
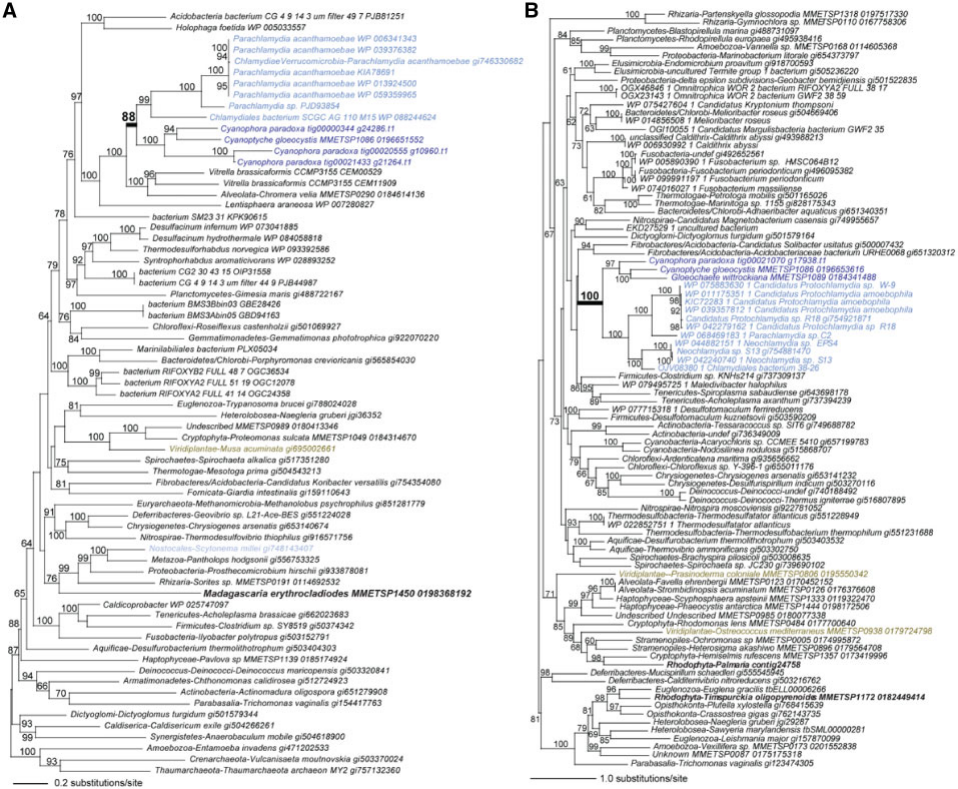
Chlamydial HGT candidates in Glaucophyta. Phylogeny of the (A) PPDK (pyruvate phosphate dikinase) gene family and (B) triose phosphate isomerase (TPI) inferred using IQ-TREE.^34^ The results of 1,000 ultrafast bootstraps^27^ are shown at the branch nodes (when ≥60%), and the legends for substitution rates on branches are shown. The PPDK tree was built using the best-fit model (LG+R6) chosen according to the Bayesian Information Criterion (BIC). The TPI tree was built using the best-fit model (LG+I+G4) chosen according to the BIC. NCBI or MMETSP identifications are shown for each of the sequence entries.

Our genome-wide analysis also strongly supports the early divergence of the red algae, with glaucophytes and Viridiplantae forming a well-supported clade (100% bootstrap support), a conclusion that was also reached in a recent study of nuclear data [351 protein dataset, maximum-likelihood and Bayesian analysis; see Fig. 1 in Brown *et al.*^52^] and of plastid data [60 proteins shared by the Archaeplastida and cyanobacteria; see Fig. 5, Supplementary S23 in Lee *et al.*^55^). This conclusion is at odds with several other studies. For example, Li *et al.*^56^ identified compositional biases among first and third positions of nucleotides in plastid amino acid codons and settled on an early divergence of glaucophytes based on protein data and plastid morphology (i.e. presence/absence of PG). They could not, however, significantly reject competing topologies. A basal divergence of glaucophytes within Archaeplastida was also found by Ponce-Toledo *et al.*^57^ in an analysis of a concatenated dataset of 97 plastid proteins that included the putative bacterial ancestor of plastids; i.e. the lineage including the freshwater species *Gloeomargarita lithophora*. This topology suggested that plastid primary endosymbiosis occurred in a freshwater environment, and glaucophytes are freshwater taxa.^9^ However, our finding of an early split of Rhodophyta within Archaeplastida does not argue against a freshwater environment for plastid endosymbiosis, because many early diverging red algal clades include freshwater taxa such as in the Compsopogonophyceae and Porphyridiophyceae.^9^

Our tree fails to provide support for the monophyly of Hacrobia (Cryptophyta +Haptophyta).^52^^,^^58^ Competing hypotheses place the haptophytes sister to the SAR (Stramenopiles +Alveolata +Rhizaria) clade^58^ or with members branching within Archaeplastida.^53^^,^^54^ Recent work suggests that the haptophytes either contain a stramenopile plastid via endosymbiosis of an ochrophyte-like alga,^59^ or they once possessed it but later lost this organelle in favour of the current haptophyte plastid via serial endosymbiosis.^60^

### 3.4. Insights into the ultrastructure of *cyanophora paradoxa*

We studied cell ultrastructure to provide a morphology-based perspective on the place of glaucophytes in the tree of life. Here, we used quick-freeze deep-etch electron microscopy (QFDEEM),^61^ a powerful technique to study the ultrastructure of microorganisms because no chemical fixatives or dehydrating procedures are needed. We focussed on the distinguishing features of *C. paradoxa*, rather than address all aspects of its cell morphology.

#### 3.4.1. Alveoli

These are a system of flattened membrane-delimited sacs/cisternae that are located beneath the cell membrane and linked together by narrow domains referred to as sutures. Alveoli are present in all studied members of the Alveolata, but absent from red and green algae and land plants. Their presence in glaucophytes, reported in several earlier studies^62^^,^^63^ represent an evolutionary enigma. Supplementary Figs S11 and S12 validate earlier studies and highlight alveolar fine structure in QFDEEM replicas of *C. paradoxa*.

#### 3.4.2. Pyrenoid

In cyanobacteria, ribulose-1,5-bisphosphate carboxylase/oxygenase (RuBisCO) localizes to encapsulated cytoplasmic inclusions referred to as carboxysomes^64^ (Supplementary Fig. S13). The glaucophyte plastid contains a central non-encapsulated pyrenoid (also termed a central body^9^) (Fig. 2; Supplementary Figs S14–S16) where RuBisCO is localized.^65^ Carboxysome/glaucophyte-pyrenoid homology was an appealing idea that was studied using genetic tools,^66^ but the pyrenoid is clearly in direct contact with the stroma, weakening the idea of a glaucophyte carboxysome. In most red and green algae, pyrenoids are traversed by one or more thylakoids (shown in Supplementary Fig. S15B for the green alga *Chlamydomonas*), but in some instances this is not the case,^67^^,^^68^ as in glaucophytes.

#### 3.4.3. Thylakoids and phycobilisomes

The glaucophyte thylakoid system has a configuration which at first glance appears to be either concentric rings, like an onion, or a spiral (Fig. 2; Supplementary Figs S15 and S16), but inspection of cross-sectioned thin-section images, such as Fig. 1 in Fathinejad *et al.*,^66^ demonstrates that the thylakoids occasionally terminate or bifurcate. Their regular separation is mediated by a system of phycobilisomes that associate with the thylakoid surfaces and interdigitate with one another, and by a rigid envelope that maintains a spherical shape (see below). The few thylakoid termini adjacent to the central pyrenoid (Supplementary Fig. S15A, arrows) lack associated phycobilisomes, are dilated, contain an osmiophilic material,^66^^,^^69^ and possibly mark the domain of thylakoid assembly; i.e. the ‘thylakoid centre’ in cyanobacteria.^70^

Giddings *et al.*^71^ published a detailed study of thylakoid and phycobilisome structure in isolated *C. paradoxa* plastids, including precise measurements of their components. Our *in vivo* images (Supplementary Figs S16–S18) largely confirm their observations. In Supplementary Fig. S16, we note the parallel between phycobilisome organization in *C. paradoxa* (Supplementary Fig. S16A) and in the red alga *Galdieria sulphuraria* (Supplementary Fig. S16B). Supplementary Fig. S17 documents a parallel between the rows of thylakoid B-face intramembranous particles in *C. paradoxa* (Supplementary Fig. S17A and C) and in the cyanobacterium *Synechococcus* 7002 (Supplementary Fig. S17B). Such rows are also present in red algae,^72^ and presumably reflect phycobilisome interactions.

#### 3.4.4. Peptidoglycan

Cyanobacteria, like all Gram-negative bacteria, assemble a layer of PG, a polymer of distinctive sugars and amino acids, between their outer and inner membranes.^73^ At the onset of division, the PG layer thickens and invaginates to form a medial furrow that constricts the inner membrane centripetally. The outer membrane follows inward, and the membranes fuse to create two daughters. In some QFDEEM images of the cyanobacterial furrow, the PG layer appears amorphous (Supplementary Fig. S19A), whereas others show the presence of filamentous material (Supplementary Fig. S19B).

Several studies have shown that glaucophytes synthesize PG^74–76^ that surrounds the plastid and extends into the furrow. Giddings *et al.*^71^ presented images of a thin smooth layer of material between the outer and inner membranes of the plastid envelope in freeze-fracture replicas of isolated organelles. Kojima *et al.*^77^ isolated PG from *C. paradoxa* and studied the plastid outer membrane proteins CppS/F that confer permeability and are of non-cyanobacterial (likely Planctomycete origin). Such material is not evident in QFDEEM cross-fractures *in situ* (Supplementary Fig. S20A) and is absent from thin-sectioned cross-sections of the moss chloroplast envelope^78^^,^^79^ which has been suggested, using a metabolic labelling ‘click chemistry’ approach, to be surrounded by PG-related material.^79^ In contrast, a wall layer is clearly present between the outer and inner membranes of the α-cyanobacterial primary endosymbiont (chromatophore) in *Paulinella chromatophore*.^80^ The surface of isolated plastids also appears to be smooth and wall-like in SEM images.^44^ Evidence that this material is PG comes from pigment release when *C. paradoxa* plastids are treated with lysozyme (56) and the finding of plastid swelling when cells are treated with penicillin.^81^

A medial invagination of the glaucophyte plastid inner membrane persists throughout interphase (Supplementary Fig. S20B), which transforms into a cleavage furrow when the plastid divides (Supplementary Fig. S20C). In contrast, medial invaginations are only seen in cyanobacteria during cell division. Material within the glaucophyte invagination appears amorphous in some images^66^^,^^71^^,^^82^^,^^83^ and includes filaments in others^44^^,^^62^^,^^69^ (Supplementary Fig. S20B and C). Further evidence for PG in the furrow rests on the localization of the PG-hydrolysing enzyme DipM to the *C. paradoxa* furrow.^84^ FtsZ ring formation is also involved in cleavage-furrow formation.^44^

The PG layer of bacterial walls is resistant to solubilization by non-ionic detergents such as NP-40. We therefore tested the integrity of the *C. paradoxa* plastid to detergent exposure. As shown in Supplementary Fig. S21, these plastids are unaffected by 1% NP-40 (as well as 5% NP-40; data not shown). In comparison, plastids of the green alga *C. reinhardtii* are fully solubilized by 0.1% NP-40.^85^

#### 3.4.5. Other cellular features

Included in our replicas are high-quality images of the *C. paradoxa* mitochondrion (Supplementary Fig. S18B) and the contractile vacuole, Golgi, and mucocysts in the cell anterior (Supplementary Fig. S22). An etched view of flagellar surfaces shows two kinds of mastigonemes (flagellar hairs), one short and flat (Fig. 2B), and the other long and narrow (Fig. 2C and D). The etched surface of the flagellar membrane shows units with a donut configuration (Fig. 2C) that are absent from the plasma membrane surface (Supplementary Figs S11A and S12A), demonstrating that in *C. paradoxa*, as in most eukaryotes,^86^ the two membranes are distinctive.

### 3.5. Evolutionary history of PG biosynthesis in glaucophytes

A detailed discussion of the composition and function of PG enzymes in glaucophytes can be found in Price *et al.*^9^ Here, we addressed phylogenetic origins of the major PG enzymes that were recovered with strong BLASTp (*E *≤* *10^−10^) support in the PacBio assembly of *C. paradoxa* to complement the analysis of PG ultrastructure and the cell cycle data. The genes (see Supplementary Table S7) we analysed come from table in Price *et al.*^9^ and comprise: PG transglycosylase/transpeptidase (PBP1, 2, and 3), two isoforms of UDP-*N*-acetylglucosamine 1-carboxyvinyltransferase (*murA*), UDP-*N*-acetylenolpyruvoylglucosamine reductase (*murB*), UDP-*N*-acetylmuramate-l-alanine ligase (*murC*), two putative glutamate racemase homologues (*murI*), a hypothetical d-Glu-adding enzyme (*murD*), UDP-*N*-acetylmuramoyl-l-alanyl-d-glutamate-2,6-diaminopimelate ligase (*murE*), a hypothetical alanine racemase (*alr*), d-alanine-d-alanine ligase (*ddl*), UDP-*N*-acetylmuramoyl-tripeptide-d-alanyl-d-alanine ligase (*murF*), phospho-*N*-acetylmuramoyl-pentapeptide-transferase (Lipid I synthesis, *mraY*), undecaprenyldiphospho-muramoylpentapeptide beta-*N*-acetylglucosaminyltransferase (Lipid II synthesis, *murG*), a hypothetical PG splitting enzyme (*dipM*), a hypothetical glucosamine-6-*P*-synthase (*glmS*), d-alanyl-d-alanine dipeptidase (*vanX*), d-Ala-d-Ala carboxypeptidase (*vanY*), and d-alanyl-d-alanine carboxypeptidase (*dacB*/PBP5). Phylogenetic analysis of individual proteins using the novel glaucophyte genomic data shows that many of them are of cyanobacterial provenance in *C. paradoxa* (e.g. PBP1/2, *murF*, and *mraY*), but a variety of different bacteria have also contributed to this pathway suggesting a history of HGT-derived gene replacement in *C. paradoxa*. We cannot, however, exclude the possibility that the plastid donor encoded this taxonomically diverse set of PG genes prior to primary endosymbiosis (Supplementary Fig. S23 and Table S7).^87^ As described above, the two homologous proteins CppS/F are PG-associated and provide a channel to allow diffusion of small substrates across the outer plastid membrane.^77^ Proteins imported into the plastid rely on the Toc-Tic-based translocon complexes, but it is not understood how they cross the intervening PG layer.

### 3.6. Analysis of chlamydial HGTs

Some of us previously proposed the ‘ménage à trois’ hypothesis (MATH) to explain the presence of the chlamydial HGT signal in Archaeplastida genomes by positing a direct role of Chlamydiales in the metabolic integration of the ancestral plastid.^88^^,^^89^ Under the MATH, Chlamydiales housed the cyanobiont inside the parasitic inclusion, thereby protecting it from host antibacterial defences. Direct conjugative transfer is postulated to have occurred within the inclusion from the chlamydial cell to the cyanobiont. The genes transferred encoded chlamydial transporters that were required either to export photosynthate (UhpC) and tryptophan (TyrP) or to import ATP in darkness. This would have created selection for the captured cyanobacterium to export carbon, amino acids, and vitamins to the inclusion lumen for pathogen benefit. Genes of tryptophan,^88^^,^^89^ isoprenoid, and menaquinone core synthesis (Cenci *et al.*, unpublished data) were also possibly transferred to support the required biochemical fluxes in the cyanobiont. In contrast, the overflow of carbon from the cyanobiont was putatively exported to the host cytosol where chlamydial enzyme effectors would have connected the tripartite symbiosis by incorporating excess carbon into host glycogen stores.^89^^,^^90^

To reassess the chlamydial contribution using the improved genome assembly from *C. paradoxa* in combination with an expanded bacterial genome database, we manually inspected the phylogenomic trees and found 35 well supported and 2 more weakly supported candidates (Supplementary Table S8). The well-supported chlamydial set includes 17 HGTs shared by all Archaeplastida lineages, whereas 3 and 5 genes are putatively shared exclusively with green and red algae, respectively. The remaining 10 genes (all present in at least 2 glaucophyte species that form a monophyletic clade and containing introns) are likely to be glaucophyte-specific HGTs. Therefore ca. 49% (likely an under-estimate due to lineage-specific gene losses that mask ancient HGT origins) of the chlamydial contribution can be traced back to the early stages of plastid endosymbiosis, prior to the radiation of Archaeplastida. Using plastid proteomics, Facchinelli *et al.*^91^ found that in contrast to other Archaeplastida, where glycolysis partitions between the plastid and cytosol,^92^*C. paradoxa* retains the full glycolytic/neoglucogenic suite of enzymes, including phosphoglyceromutase (PGAM) and enolase, within the plastid stroma. This result is consistent with the paucity of candidate transporters in the *C. paradoxa* plastid membrane, specifically one that can translocate phosphoenolpyruvate (PEP). In this context, our finding of glaucophyte-specific chlamydial HGT candidates with respect to PPDK (pyruvate phosphate dikinase; Fig. 5A) that gave rise to a gene family in this species and triose phosphate isomerase (TPI; Fig. 5B) may reflect the ancestral transfer of genes required to boost anaerobic glycolysis in the nascent plastid. Previous experimental work using *Entamoeba histolytica* recombinant enzymes^93^ showed that TPI, PGAM, and PPDK have the highest (i.e. with respect to other pH) control coefficient values at pH 7 for anaerobic glycolysis, and PGAM is a well-supported chlamydial HGT candidate in the green lineage.^90^

Pyrophosphate-dependent anaerobic glycolysis enzymes are absent from cyanobacteria Gloeobacterales *sensu lato* (inclusive of *G. lithophora*). Early implementation of such a pathway during plastid endosymbiosis would have relieved ATP starvation under hypoxia in darkness because the cyanobacterial endosymbiont was likely exporting large amounts of photosynthetic carbon (G6P) and energy-costly compounds such as vitamin K and tryptophan. Of relevance here is the finding of bacterial-derived GTP-dependent phosphoenolpyruvate carboxykinase (PEPCK) and carbonic anhydrase (CA) to Glaucophyta (Supplementary Fig. S24). In the presence of high CA activity, PEPCK is known in bacteria to fix CO_2_ into PEP to generate GTP and OAA^94^), which could have been further metabolized at the early stages of plastid endosymbiosis by menaquinone-driven respiration in the microaerophilic environment of the chlamydial inclusion.^90^ The coupling of anaerobic glycolysis with ‘microaerophilic’ respiration would have been an efficient way to maintain ATP homeostasis in darkness under hypoxic stress, avoiding ATP hydrolysis and maximizing the use of pyrophosphate produced by other pathways. The idea that ATP starvation in darkness was a major stress experienced by the incipient cyanobacterium at the onset of plastid primary endosymbiosis is consistent with the chlamydial derivation of the ATP/ADP translocase in the Archaeplastida ancestor.^95^

Two types of HGT signal would result from the proposed tripartite symbiosis under the MATH. The first is analogous to the expected HGTs from different bacterial sources during the process of organelle DNA degeneration. These genes were needed to resurrect plastid pathways, such as for the biosynthesis of different amino acids (e.g. *Paulinella chromatophora*^95^). The second HGT signal would reflect the ancient cyanobacteria/pathogen biotic interaction. These genes, present on the chimeric plastid genome, would have their frequency of transfer boosted by EGT rather than HGT, in particular when the pathways or transporters were not initially encoded by the cyanobacterium. We postulate that HGT/EGT of the genes encoding UhpC, NTT, and TyrP transporters, as well the genes of tryptophan, isoprenoid, and menaquinone biosynthesis, represent such ancient biotic interactions.^89^ This hypothesis is supported by the observation of multiple chlamydial transfers in these pathways, most of which are shared by all three Archaeplastida lineages. The idea is also consistent with the finding of chlamydial HGTs that encode transporters required to export the final products of the proposed biotic interactions (e.g. photosynthetic carbon, tryptophan) in the proposed inclusion. Finally, these ideas are also strengthened by our current knowledge of chlamydial biology concerning, for instance, the recognized central importance of tryptophan metabolism and hypoxia in the chlamydial replication cycle.^96^

Therefore, we suggest that anaerobic glycolysis under control of PiPi-dependent phosphofructokinase, TPI, PGAM, PPDK, CA, and GTP-dependent PEPCK, whose HGT/EGTs from Chlamydiae and other bacteria are suggested in this work, may reflect an ancient biotic interaction. This process resulted in the intracellular transfer of these genes to the ancestral cyanobacterium due to its location in the chlamydial inclusion. The Glaucophyta are of particular interest under the MATH because unlike the distinct cytosol/plastid partitioning of glycolysis in the red and green lineages, *C. paradoxa* appears to represent the ancestral condition of maintaining all these functions in the plastid.

## 4. Conclusive remarks

In summary, the significantly improved genome assembly presented and analysed here provides several important insights. First, the phylogenetic analysis of a partitioned 3,000 OG dataset from 115 eukaryote proteomes that includes the new *C. paradoxa* gene models provides robust support for Archaeplastida monophyly. This analysis also supports with high bootstrap values most of the other existing supergroups. Noteworthy aspects of the *C. paradoxa* genome include its gene richness and low repeat composition with a relatively small contribution made by LTRs and LINEs. The finding of shared overlapping UTRs may indicate selection in at least one instance to co-localize functionally related genes. Any putative functional role of accessory ORFs encoded within the transcripts of *C. paradoxa* awaits further investigation. The ultrastructure work underlines many well-accepted aspects of glaucophyte cell morphology. Finally, the inventory of chlamydial and other bacterial HGTs in key pathways provides a novel lens to address the ancient biotic interactions driven by plastid primary endosymbiosis. In summary, our work further underlines the importance of *C. paradoxa* as a model species for understanding ancient events in algal evolution. Improved methods of genome sequencing and assembly provide the impetus to analyse additional glaucophyte genomes to discover the extent of divergence and footprints of ancient events driven by endosymbiosis in the ancestor of these taxa.

## Supplementary Material

dsz009_Supplementary_Data
