# Supplementary figures and images for: Analysis of an improved Cyanophora paradoxa genome assembly
Source: DNA Res. 2019 May 16;26(4):287–99. doi: 10.1093/dnares/dsz009 (PMC6704402; doi:10.1093/dnares/dsz009)

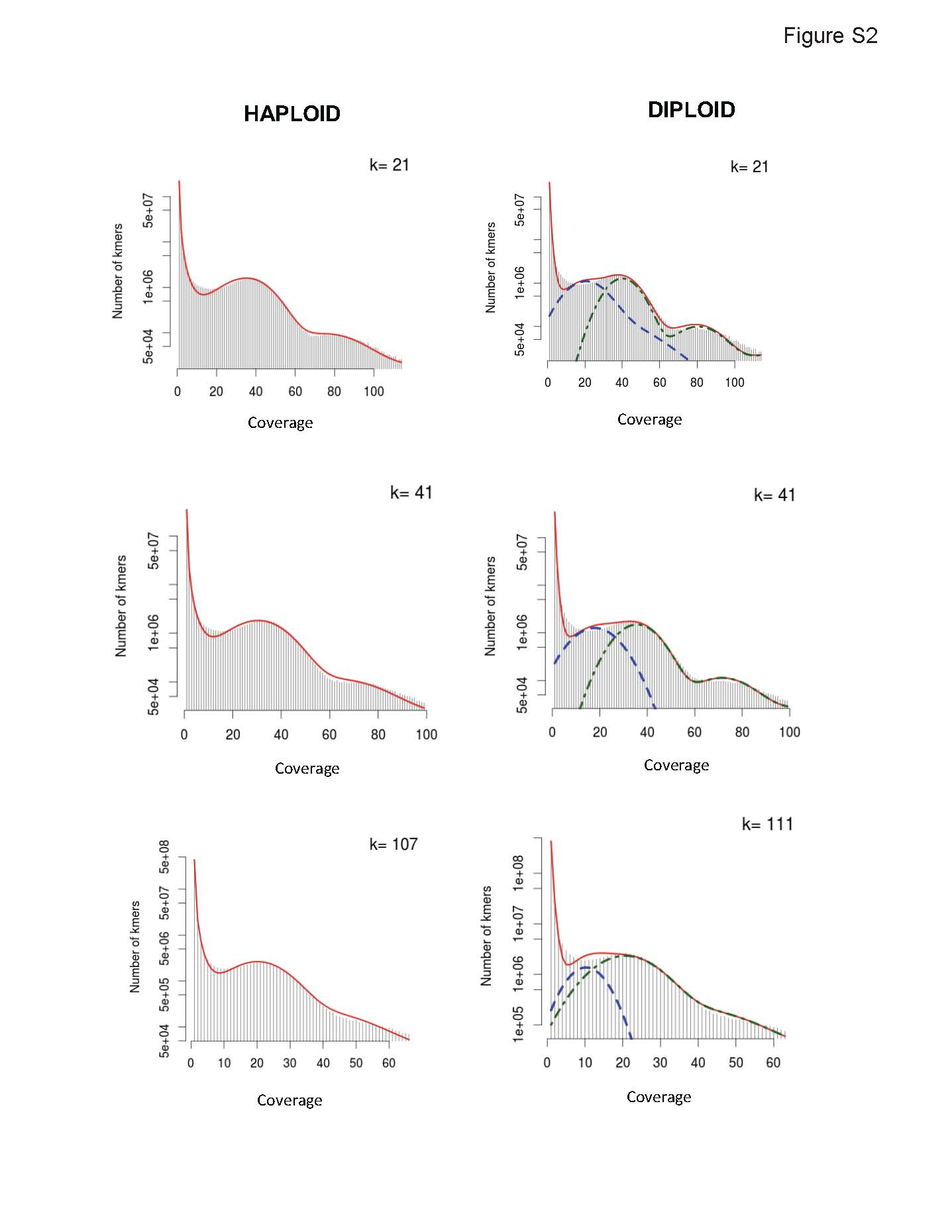

Supplement: dsz009_Supplementary_Data [file dsz009_supplementary_data.zip › dsz009-Suppl_data/Supplementary figures_Page_02.jpg]

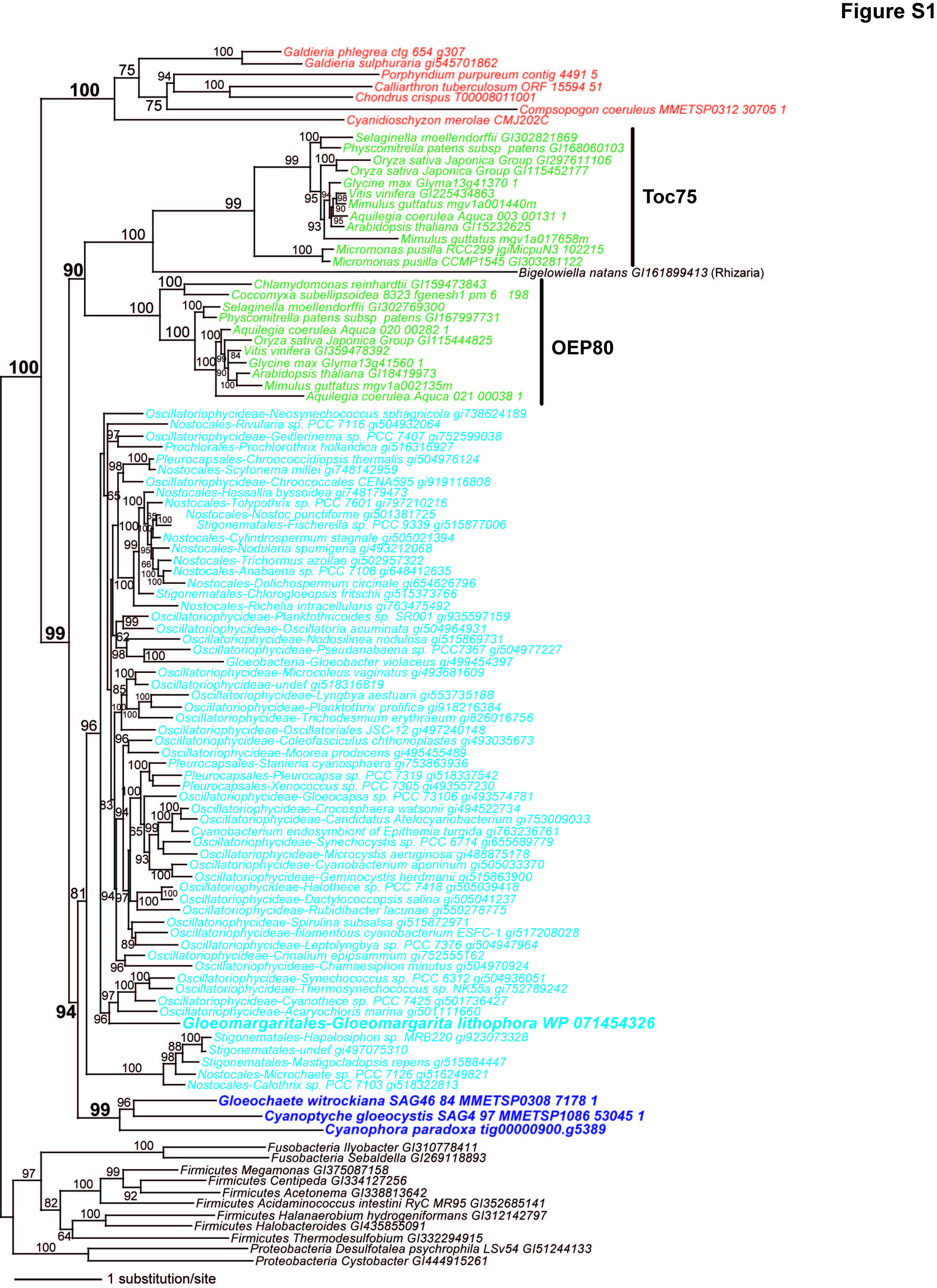

Supplement: dsz009_Supplementary_Data [file dsz009_supplementary_data.zip › dsz009-Suppl_data/Supplementary figures_Page_01.jpg]

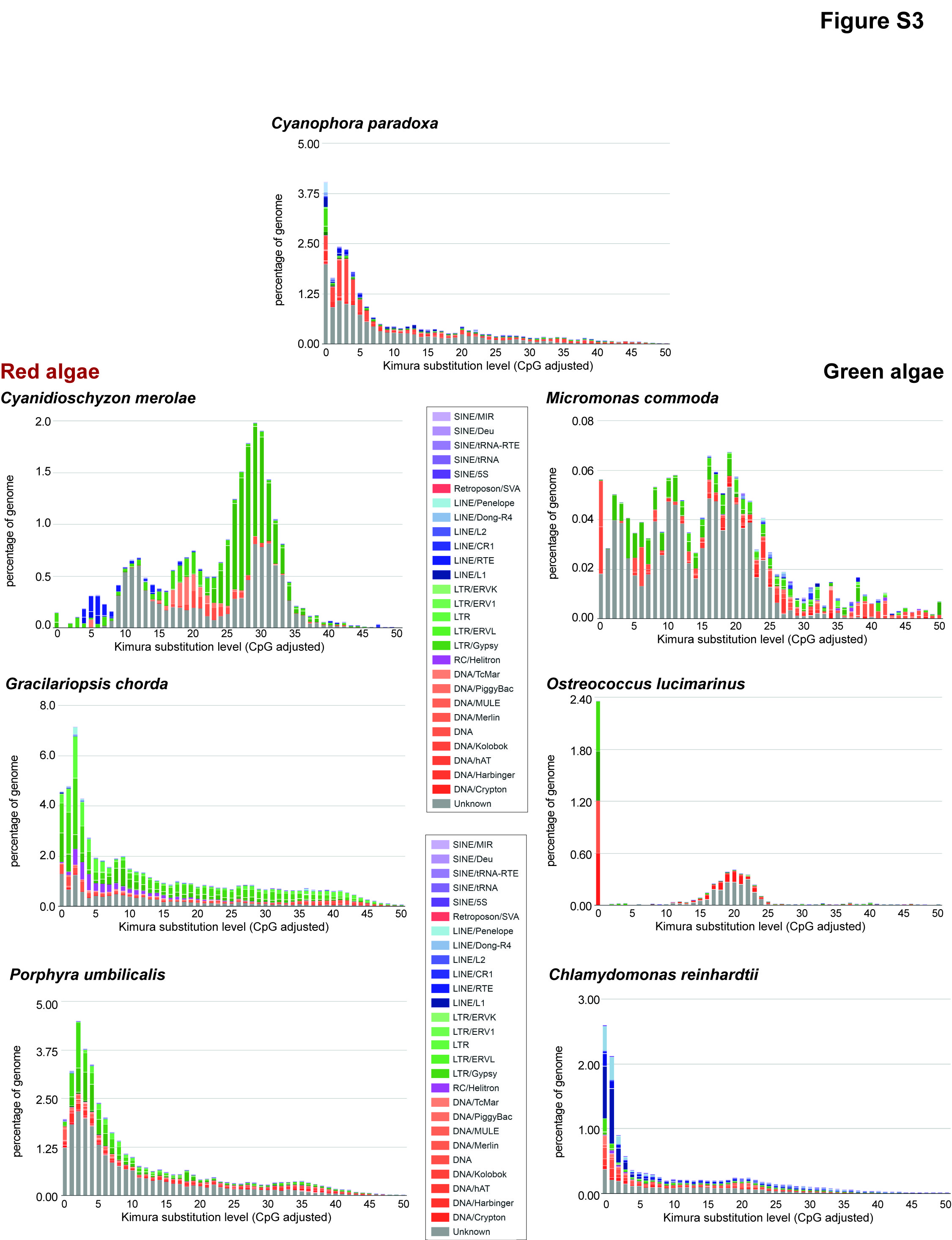

Supplement: dsz009_Supplementary_Data [file dsz009_supplementary_data.zip › dsz009-Suppl_data/Supplementary figures_Page_03.jpg]

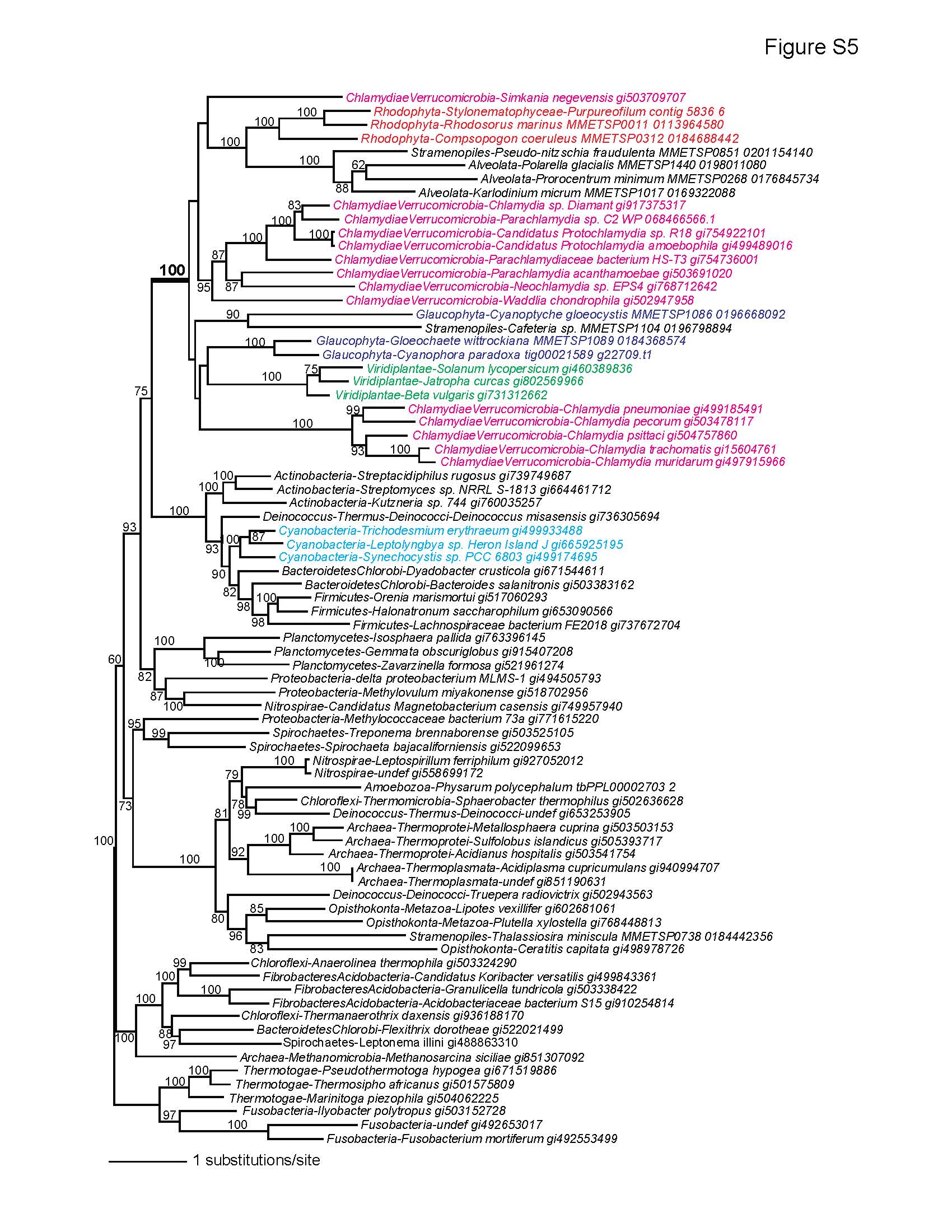

Supplement: dsz009_Supplementary_Data [file dsz009_supplementary_data.zip › dsz009-Suppl_data/Supplementary figures_Page_05.jpg]

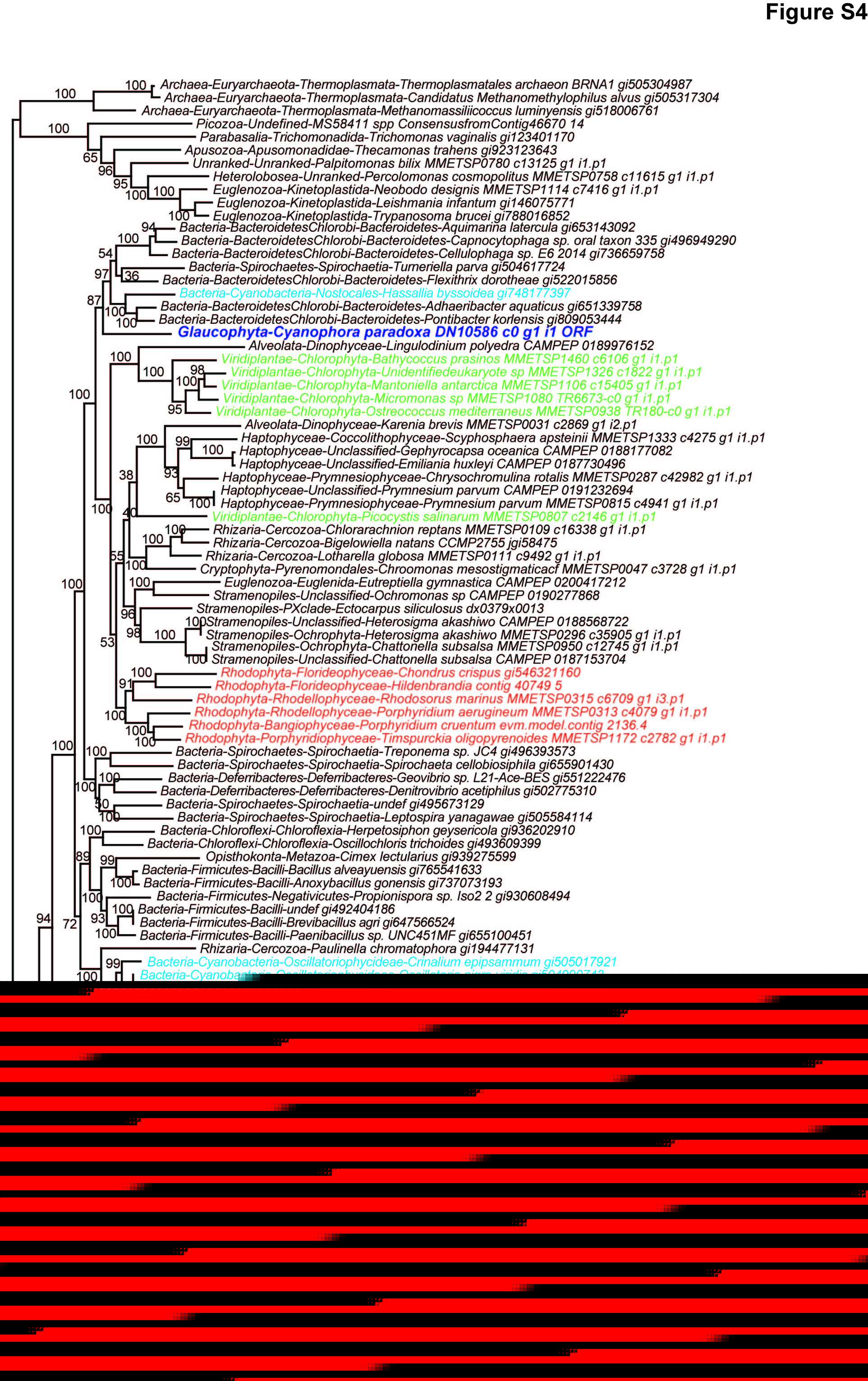

Supplement: dsz009_Supplementary_Data [file dsz009_supplementary_data.zip › dsz009-Suppl_data/Supplementary figures_Page_04.jpg]

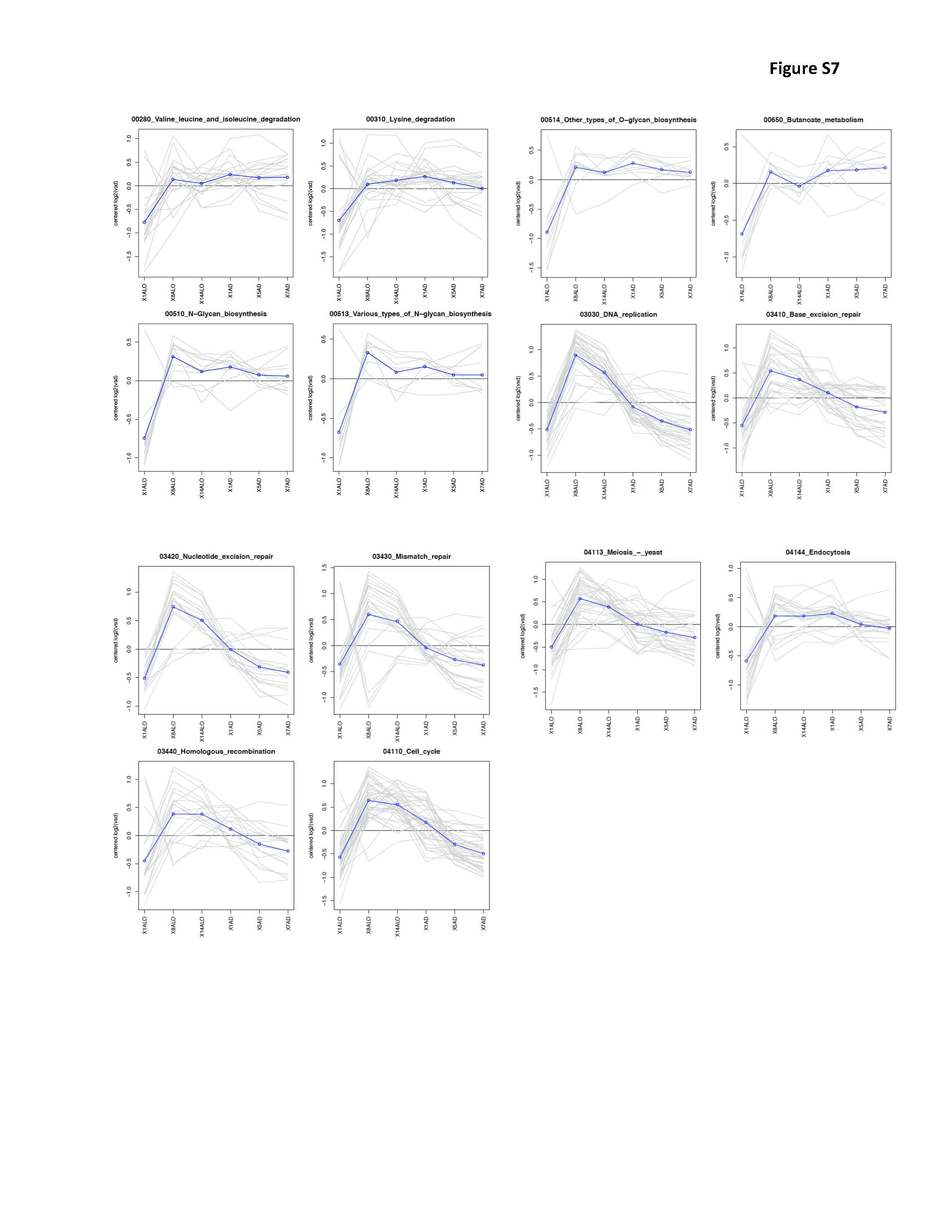

Supplement: dsz009_Supplementary_Data [file dsz009_supplementary_data.zip › dsz009-Suppl_data/Supplementary figures_Page_07.jpg]

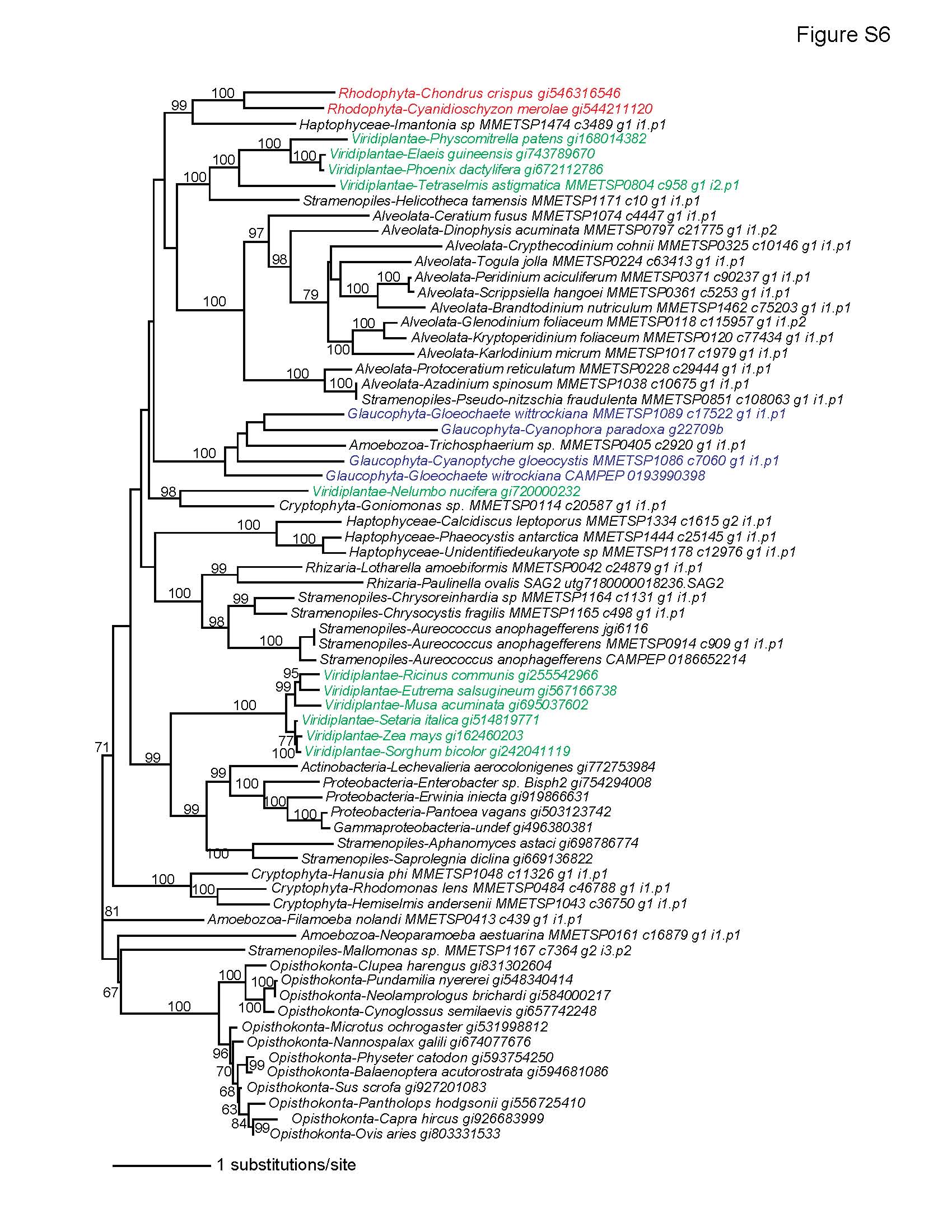

Supplement: dsz009_Supplementary_Data [file dsz009_supplementary_data.zip › dsz009-Suppl_data/Supplementary figures_Page_06.jpg]

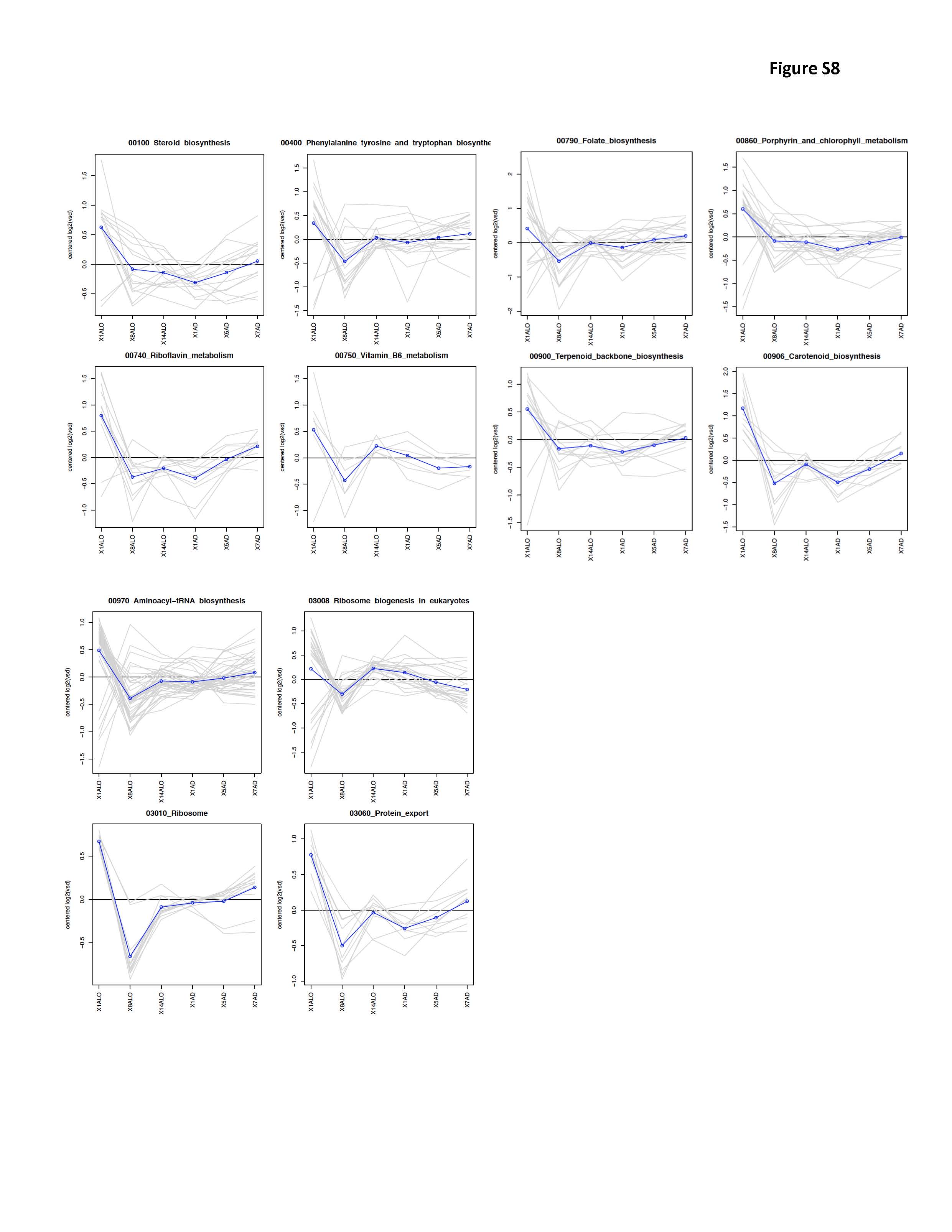

Supplement: dsz009_Supplementary_Data [file dsz009_supplementary_data.zip › dsz009-Suppl_data/Supplementary figures_Page_08.jpg]

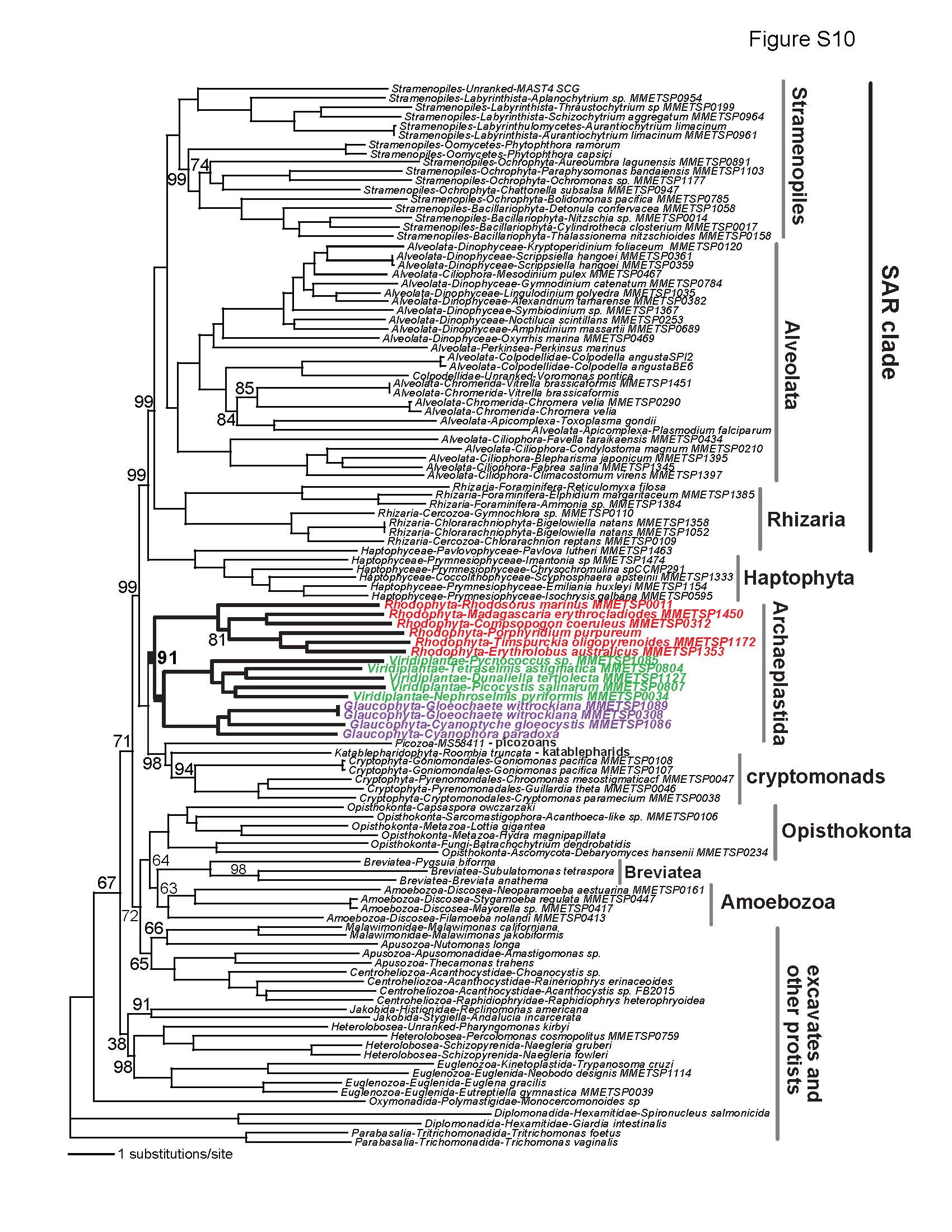

Supplement: dsz009_Supplementary_Data [file dsz009_supplementary_data.zip › dsz009-Suppl_data/Supplementary figures_Page_10.jpg]

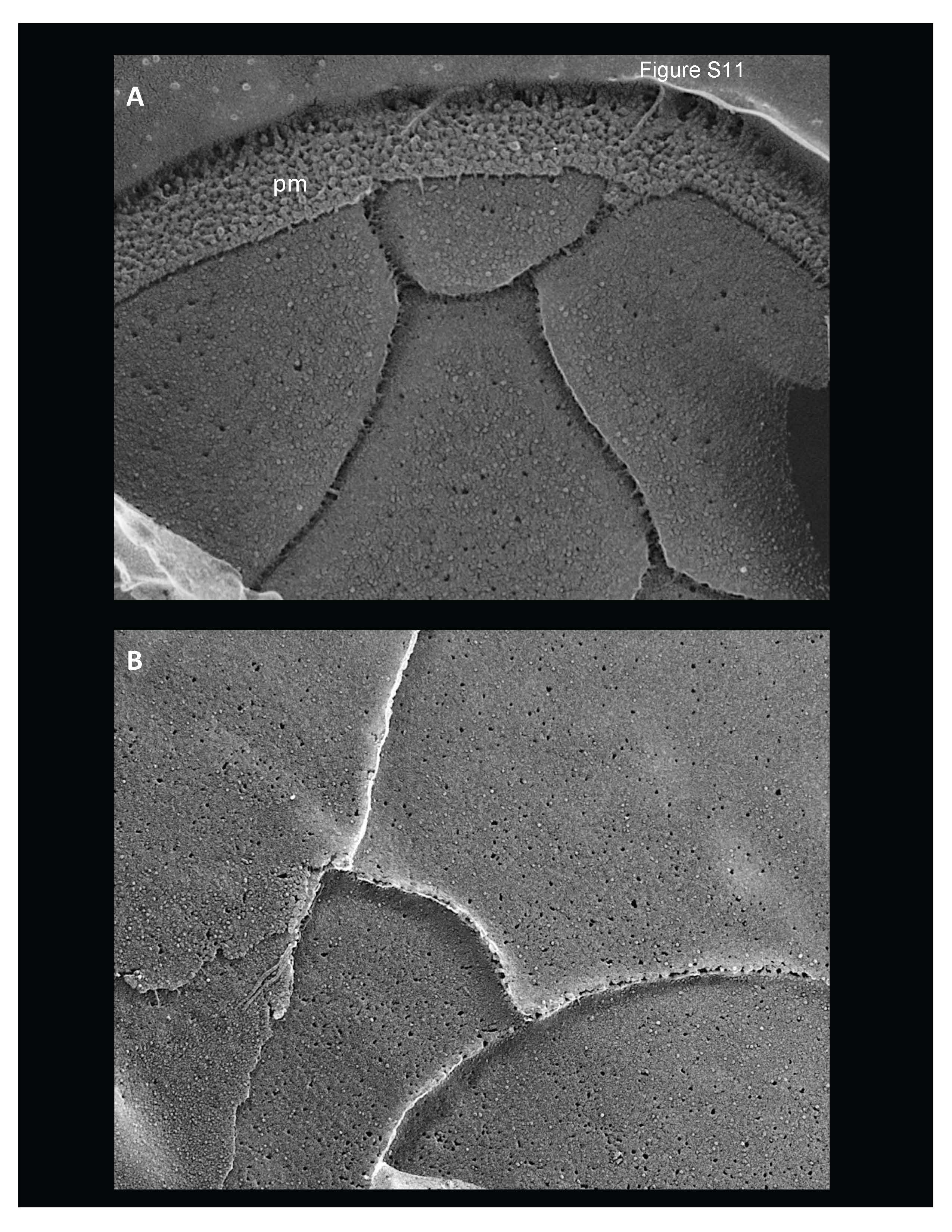

Supplement: dsz009_Supplementary_Data [file dsz009_supplementary_data.zip › dsz009-Suppl_data/Supplementary figures_Page_11.jpg]

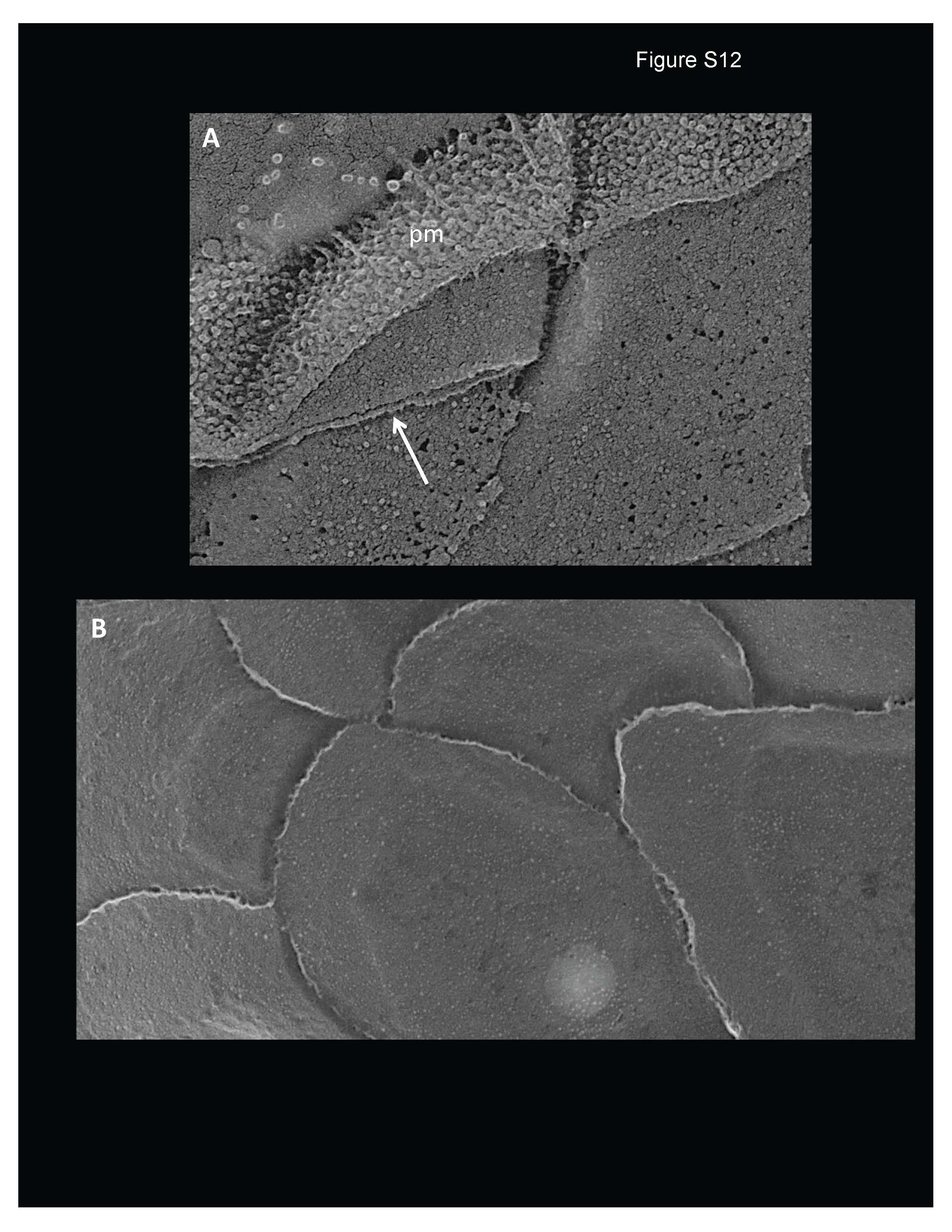

Supplement: dsz009_Supplementary_Data [file dsz009_supplementary_data.zip › dsz009-Suppl_data/Supplementary figures_Page_12.jpg]

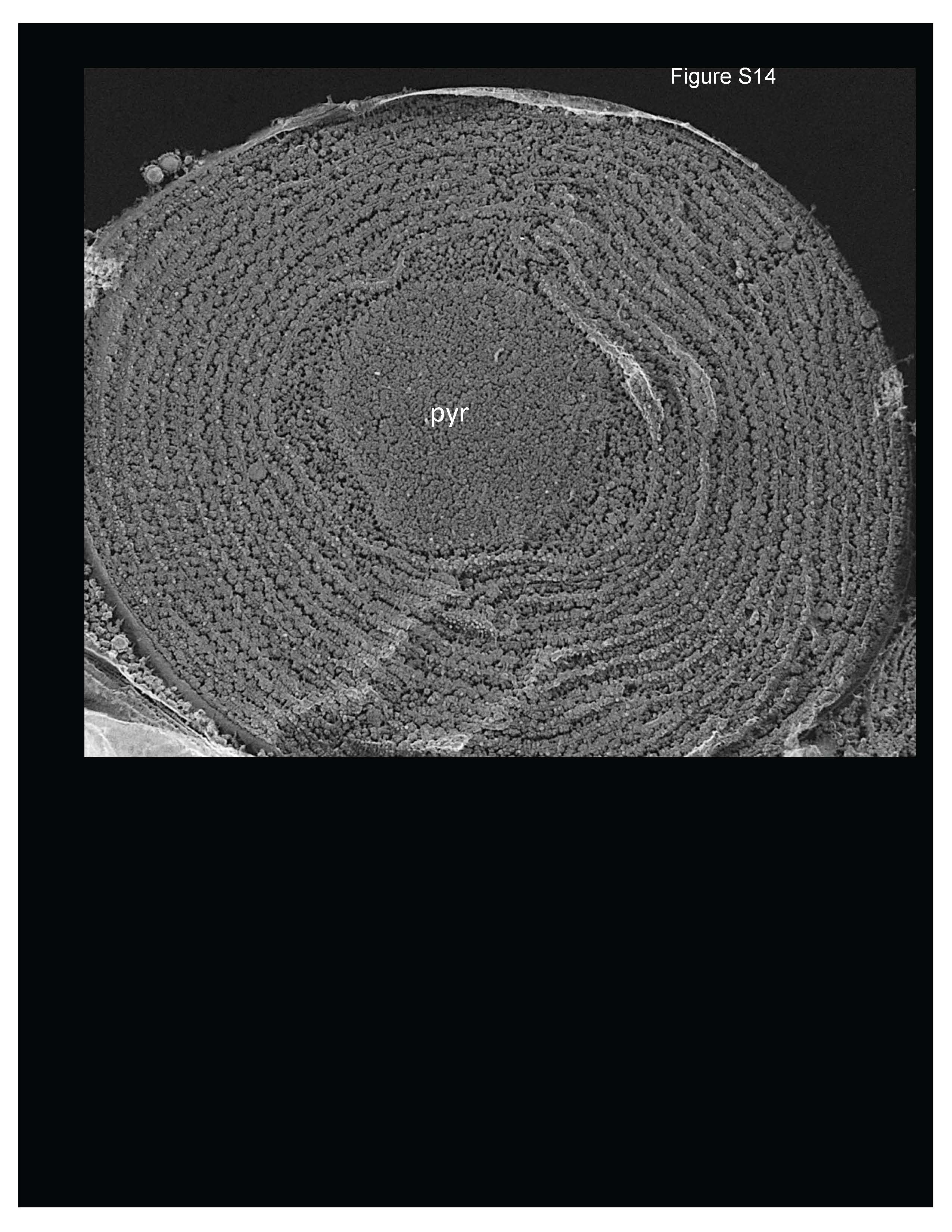

Supplement: dsz009_Supplementary_Data [file dsz009_supplementary_data.zip › dsz009-Suppl_data/Supplementary figures_Page_14.jpg]

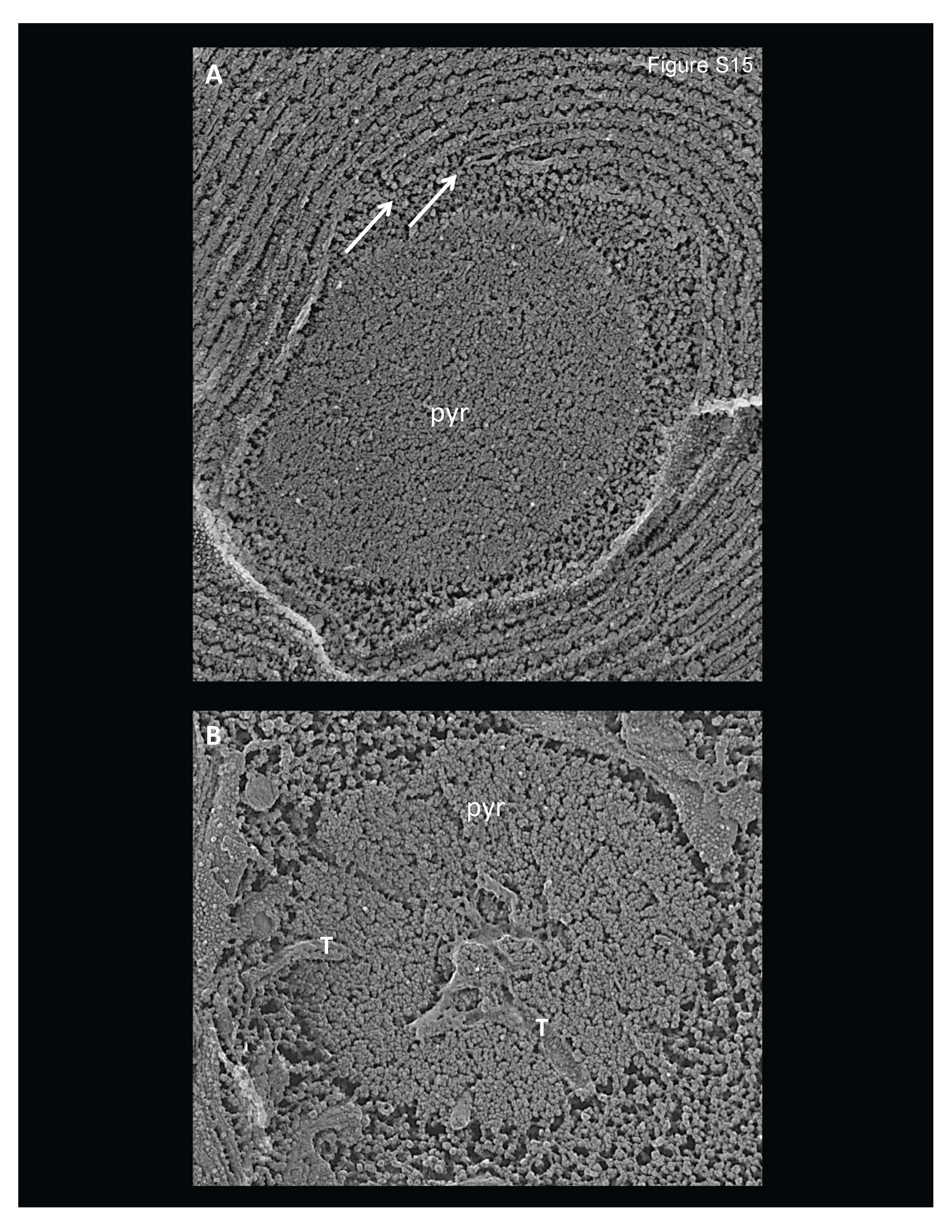

Supplement: dsz009_Supplementary_Data [file dsz009_supplementary_data.zip › dsz009-Suppl_data/Supplementary figures_Page_15.jpg]

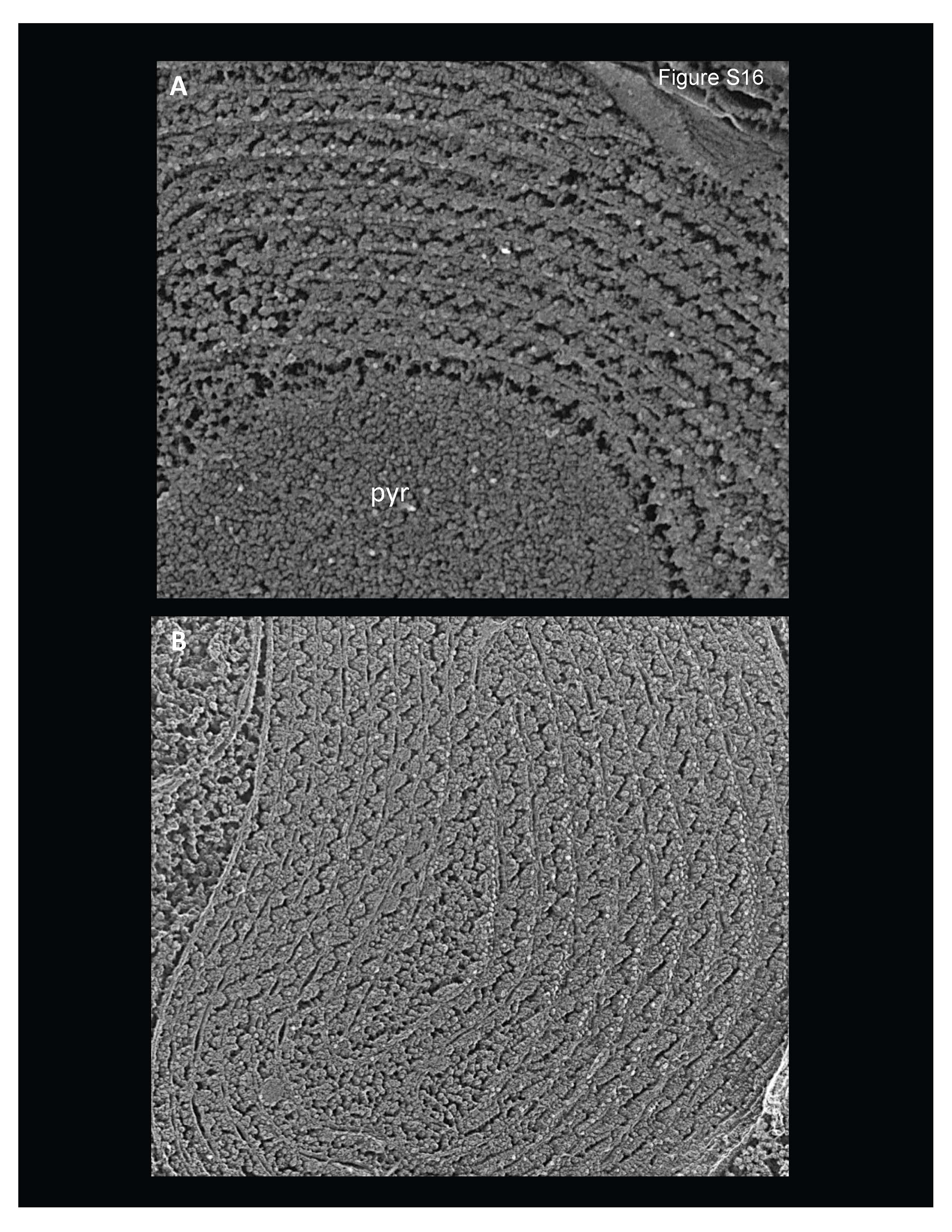

Supplement: dsz009_Supplementary_Data [file dsz009_supplementary_data.zip › dsz009-Suppl_data/Supplementary figures_Page_16.jpg]

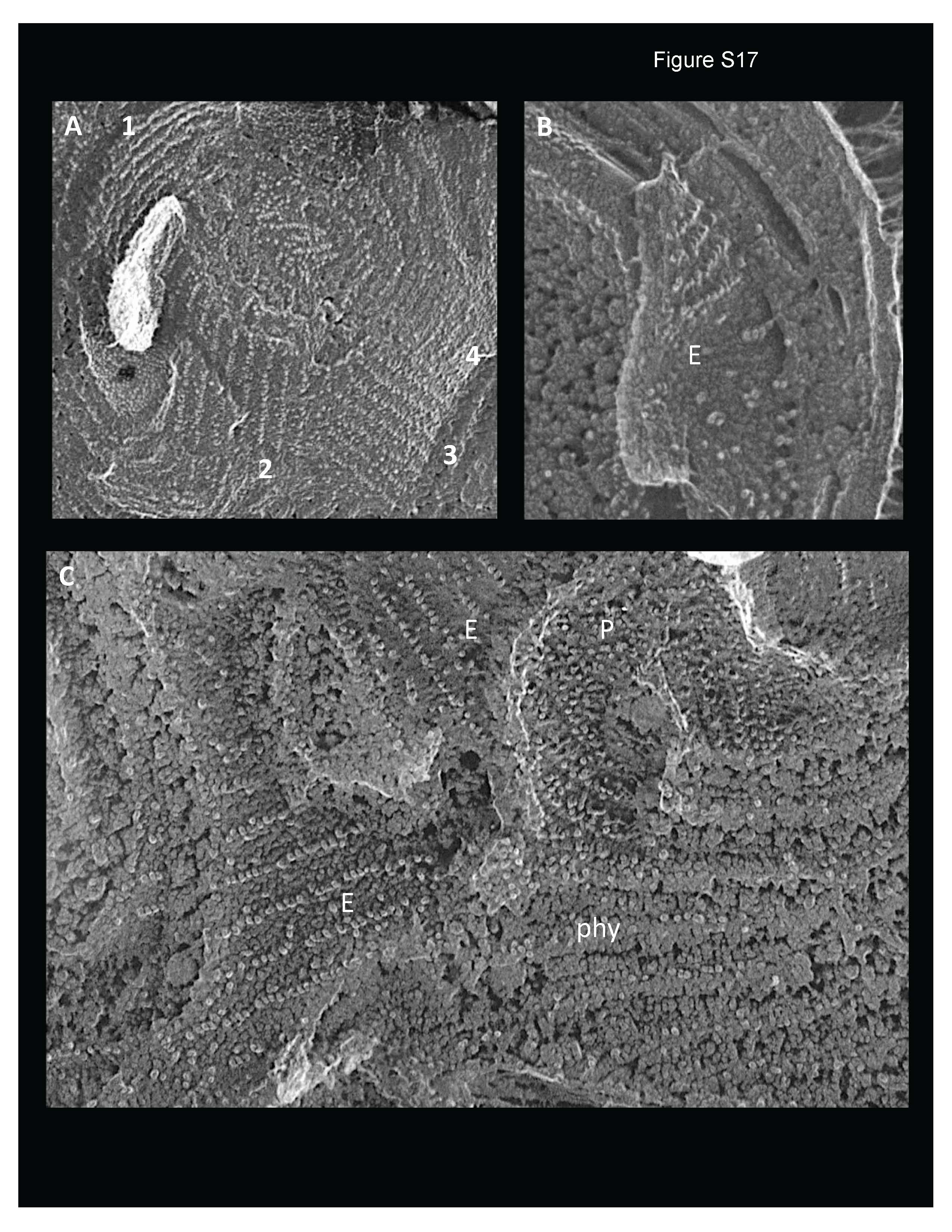

Supplement: dsz009_Supplementary_Data [file dsz009_supplementary_data.zip › dsz009-Suppl_data/Supplementary figures_Page_17.jpg]

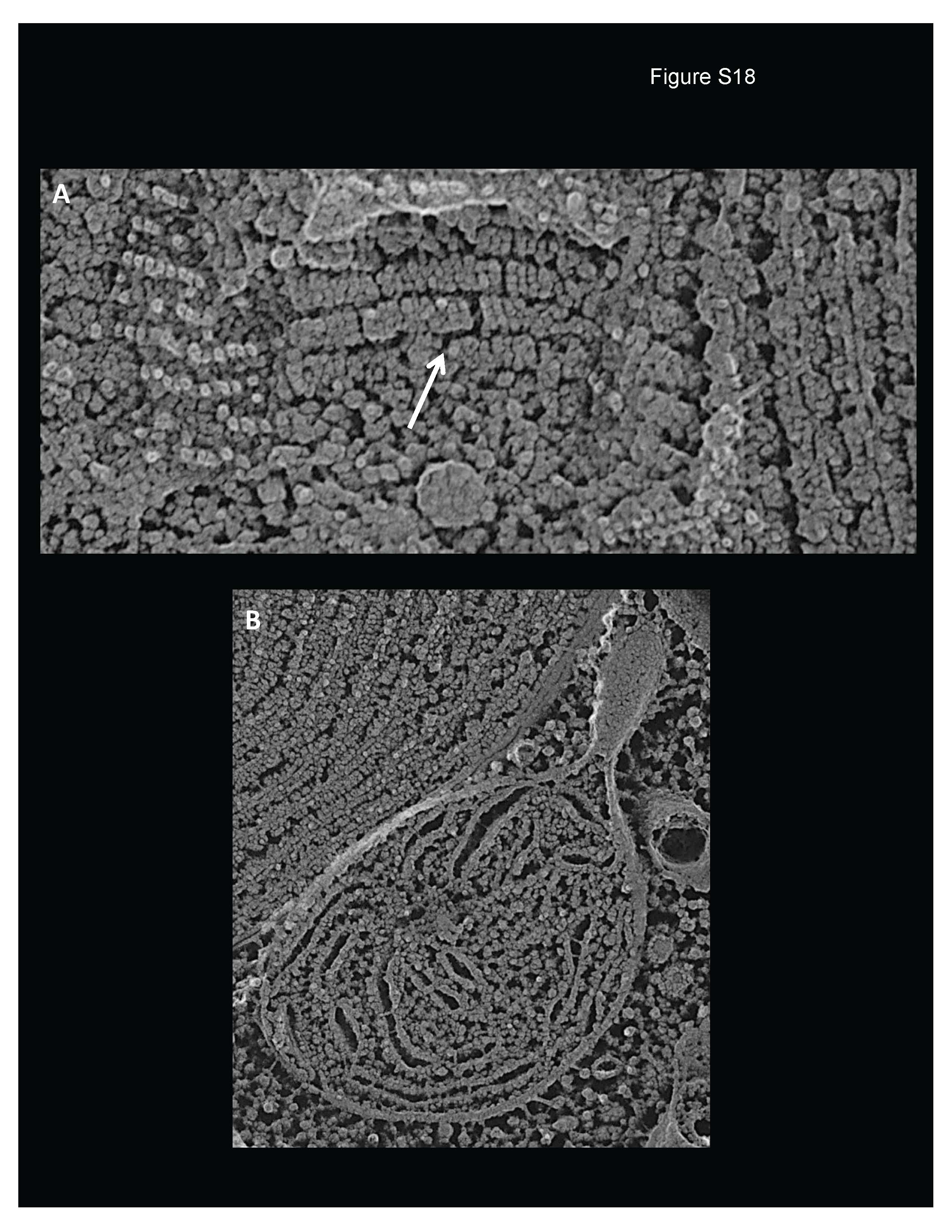

Supplement: dsz009_Supplementary_Data [file dsz009_supplementary_data.zip › dsz009-Suppl_data/Supplementary figures_Page_18.jpg]

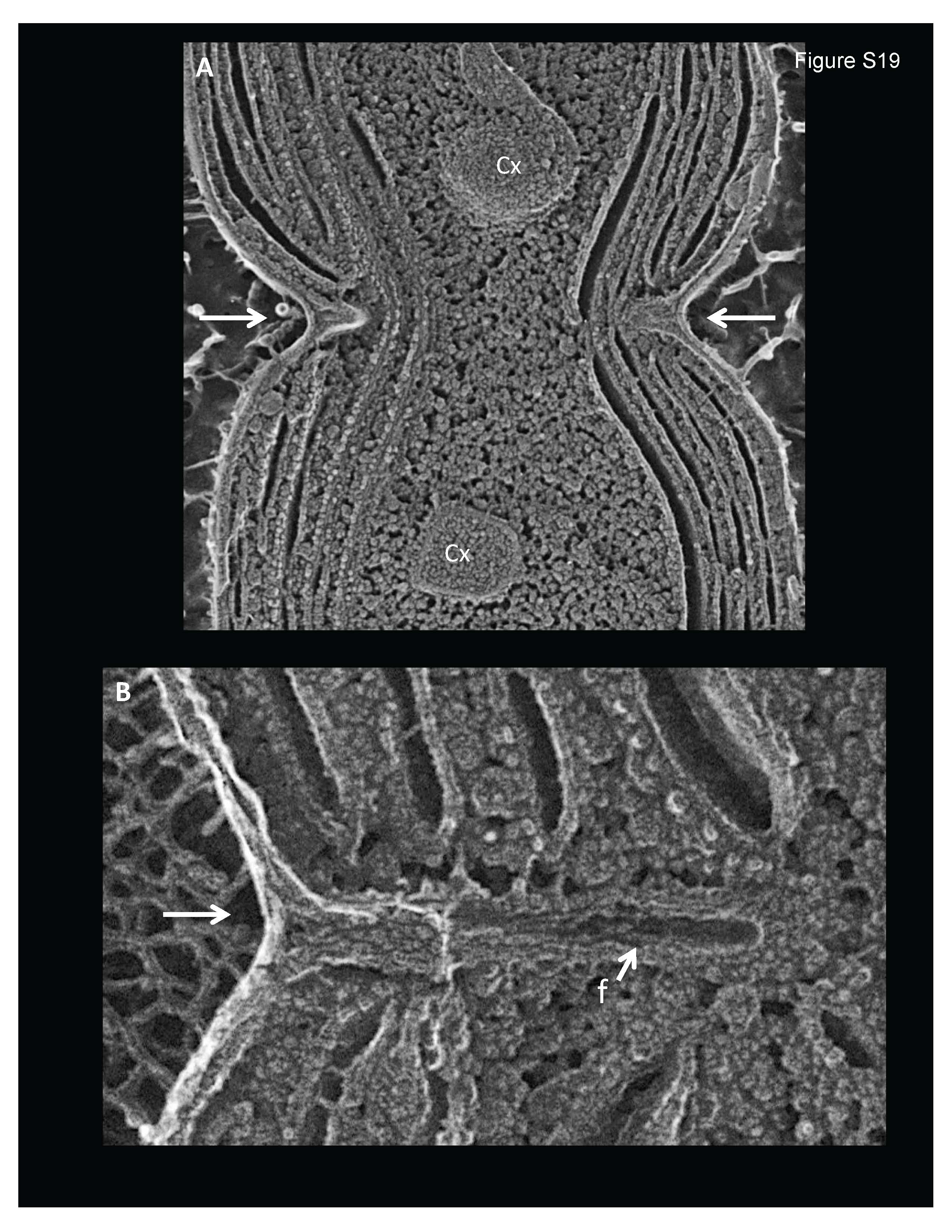

Supplement: dsz009_Supplementary_Data [file dsz009_supplementary_data.zip › dsz009-Suppl_data/Supplementary figures_Page_19.jpg]

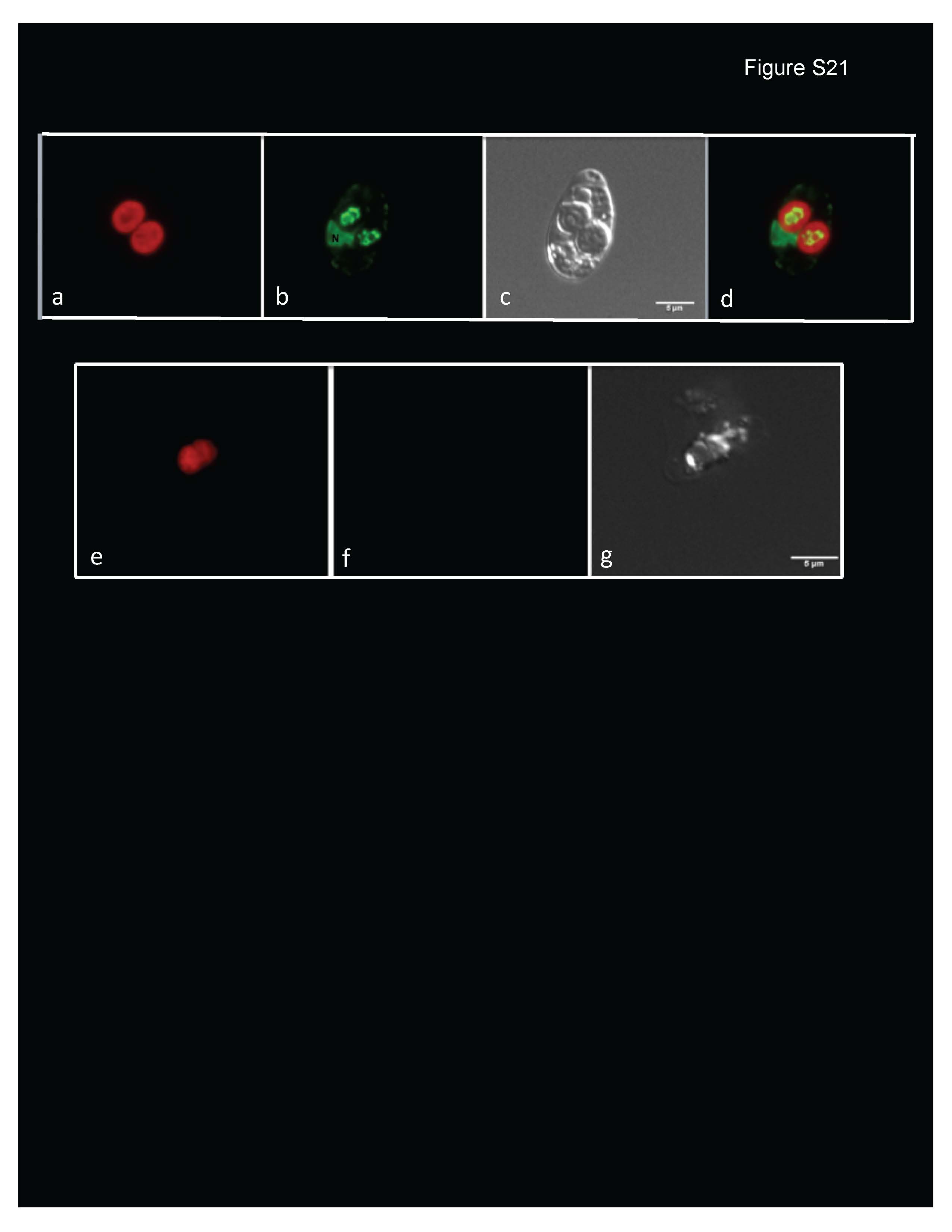

Supplement: dsz009_Supplementary_Data [file dsz009_supplementary_data.zip › dsz009-Suppl_data/Supplementary figures_Page_21.jpg]

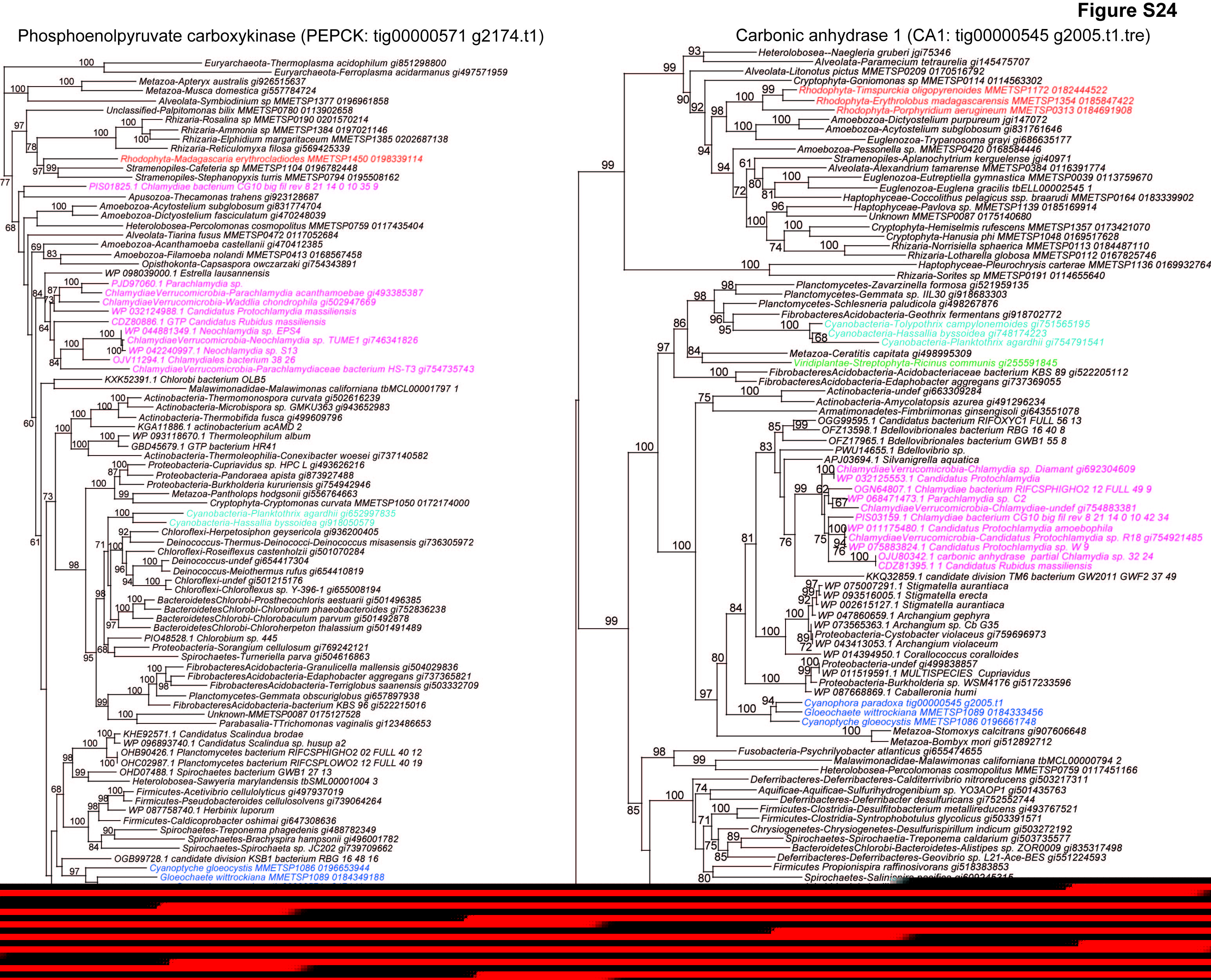

Supplement: dsz009_Supplementary_Data [file dsz009_supplementary_data.zip › dsz009-Suppl_data/Supplementary figures_Page_24.jpg]

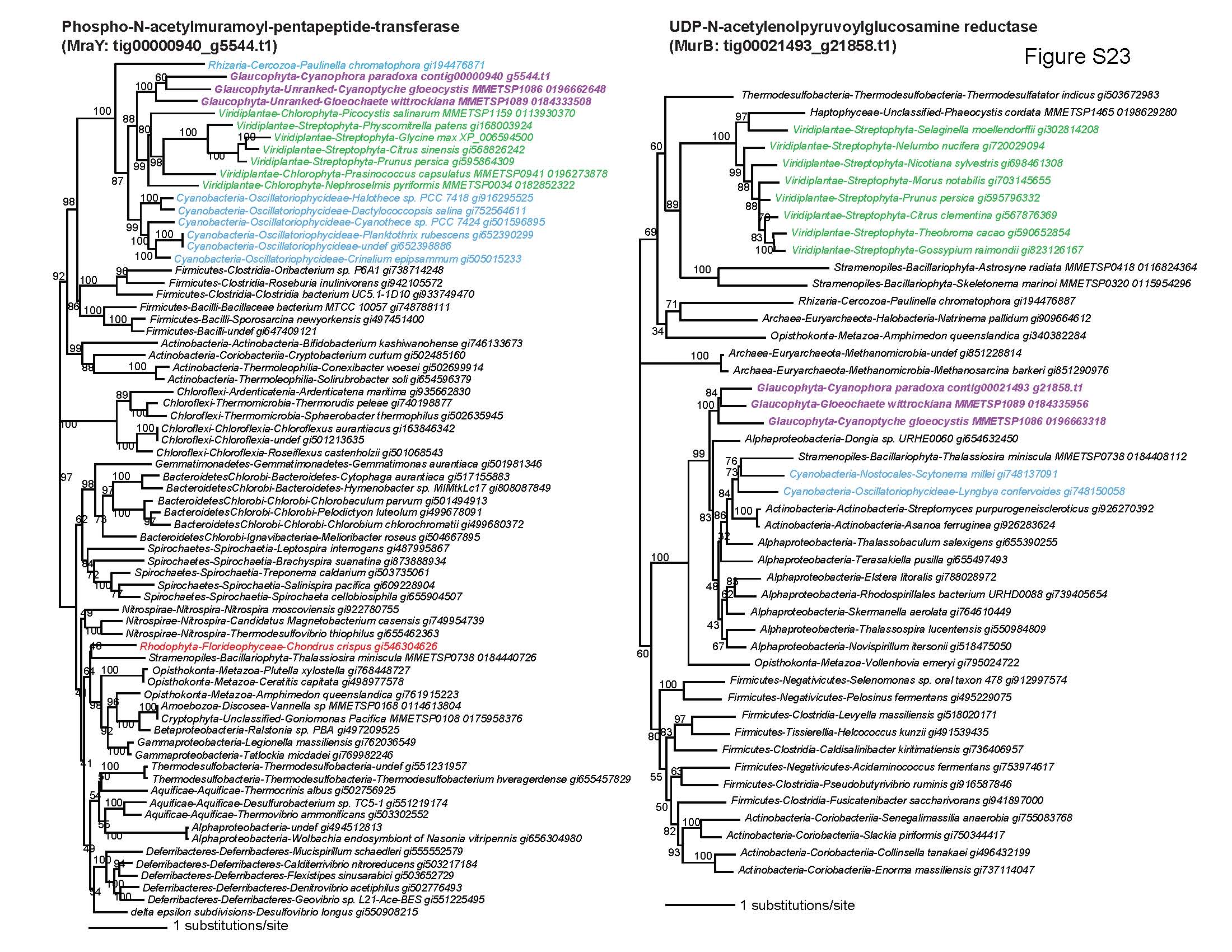

Supplement: dsz009_Supplementary_Data [file dsz009_supplementary_data.zip › dsz009-Suppl_data/Supplementary figures_Page_23.jpg]

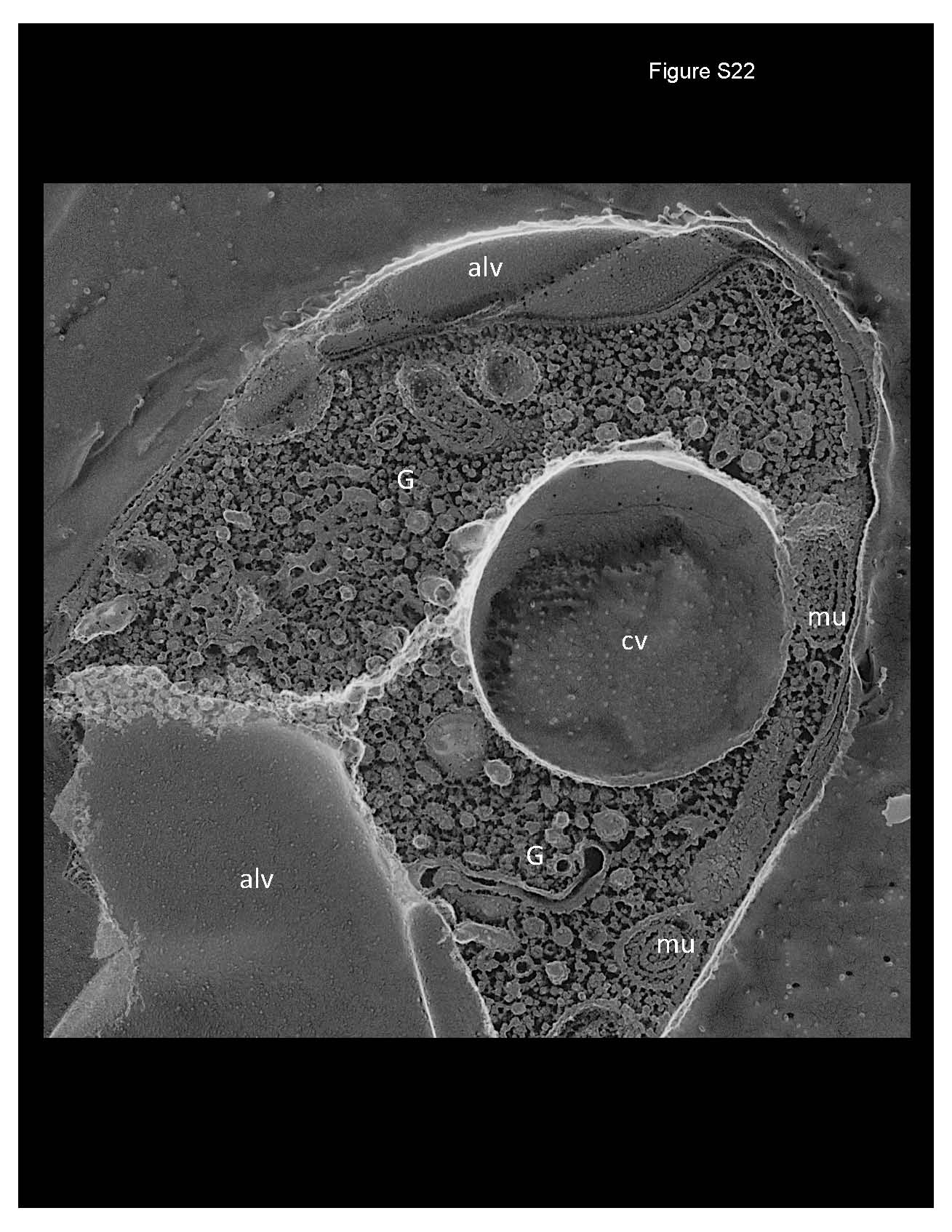

Supplement: dsz009_Supplementary_Data [file dsz009_supplementary_data.zip › dsz009-Suppl_data/Supplementary figures_Page_22.jpg]

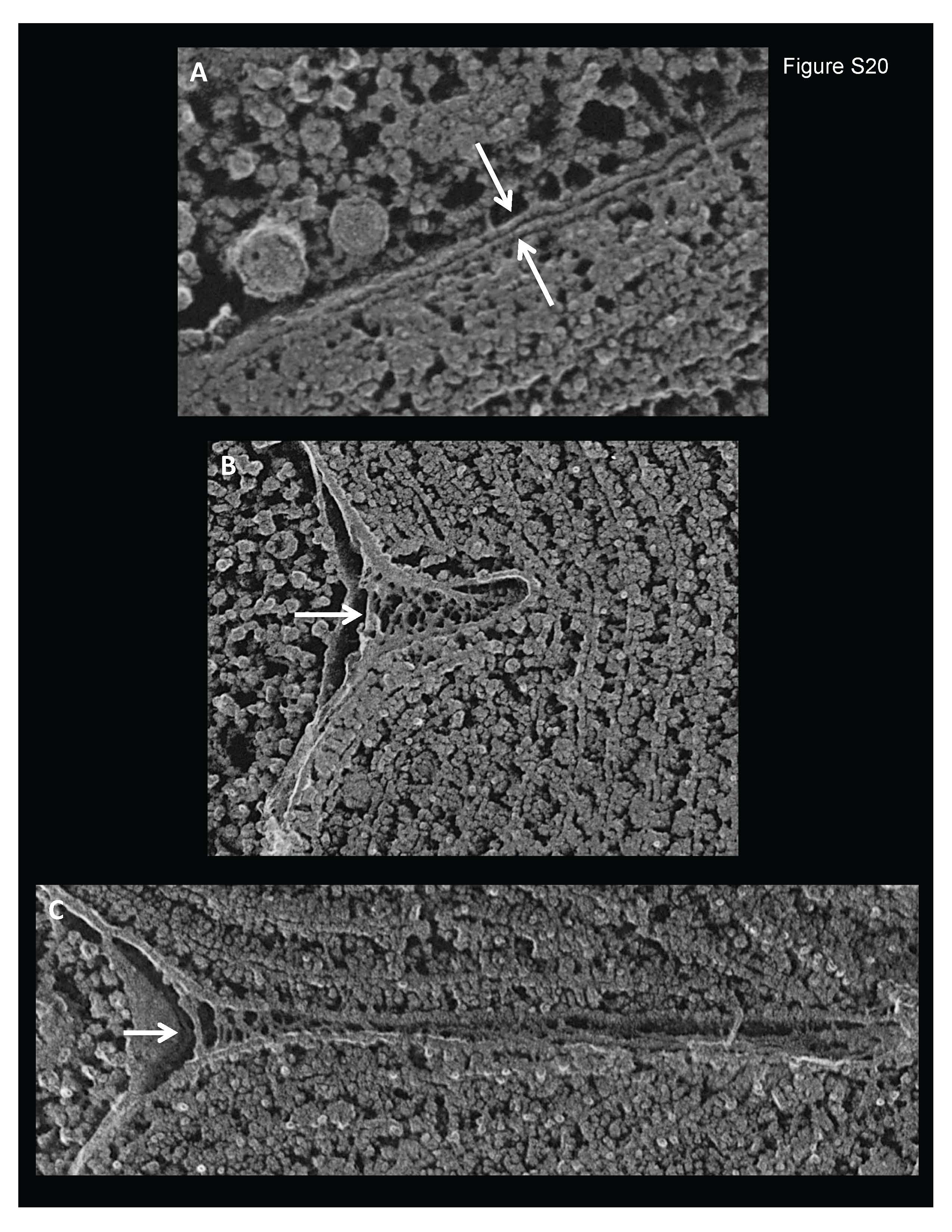

Supplement: dsz009_Supplementary_Data [file dsz009_supplementary_data.zip › dsz009-Suppl_data/Supplementary figures_Page_20.jpg]

Figure S9

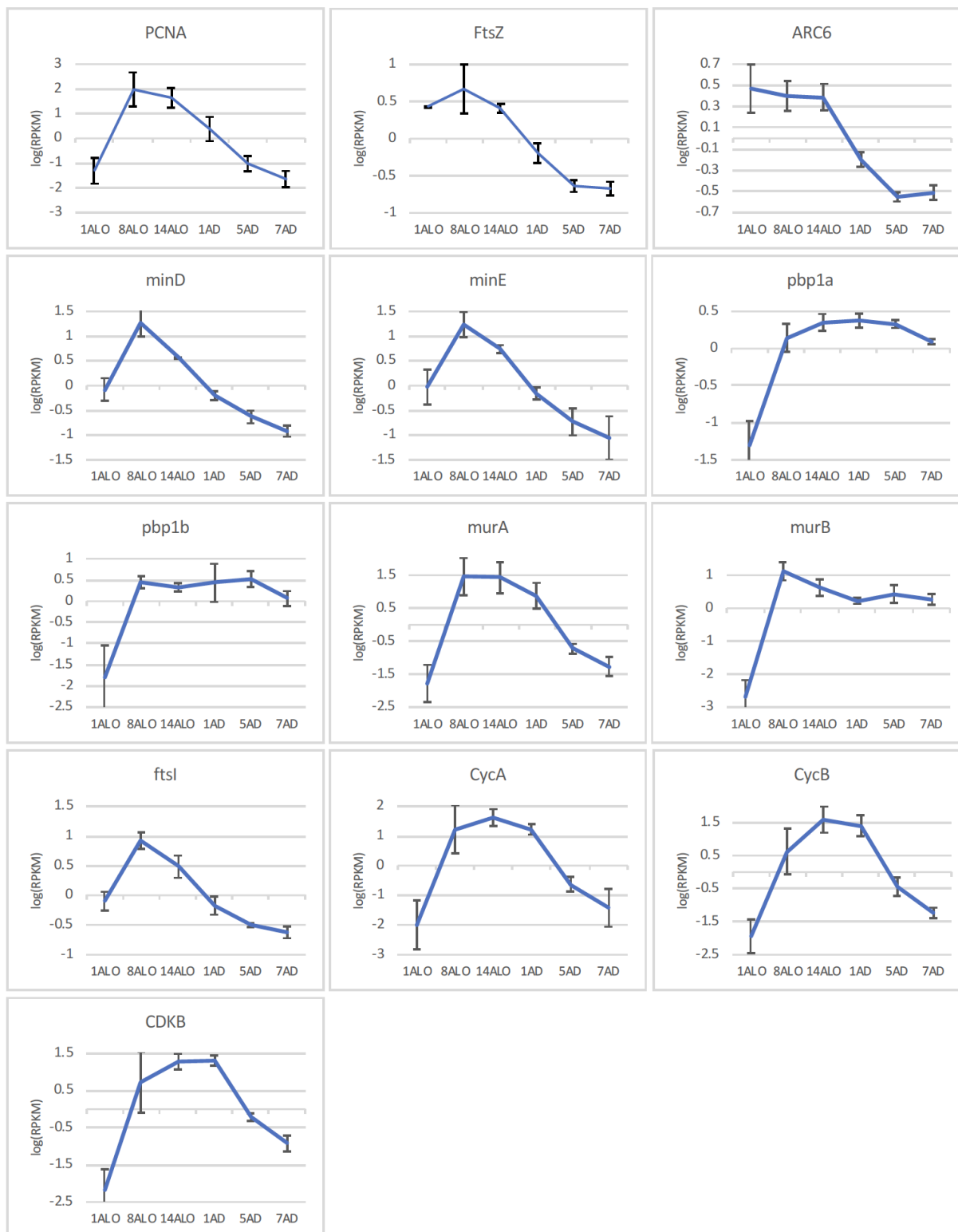

Supplement: dsz009_Supplementary_Data [file dsz009_supplementary_data.zip › dsz009-Suppl_data/Supplementary_Figure S9.pdf]

Figure S13

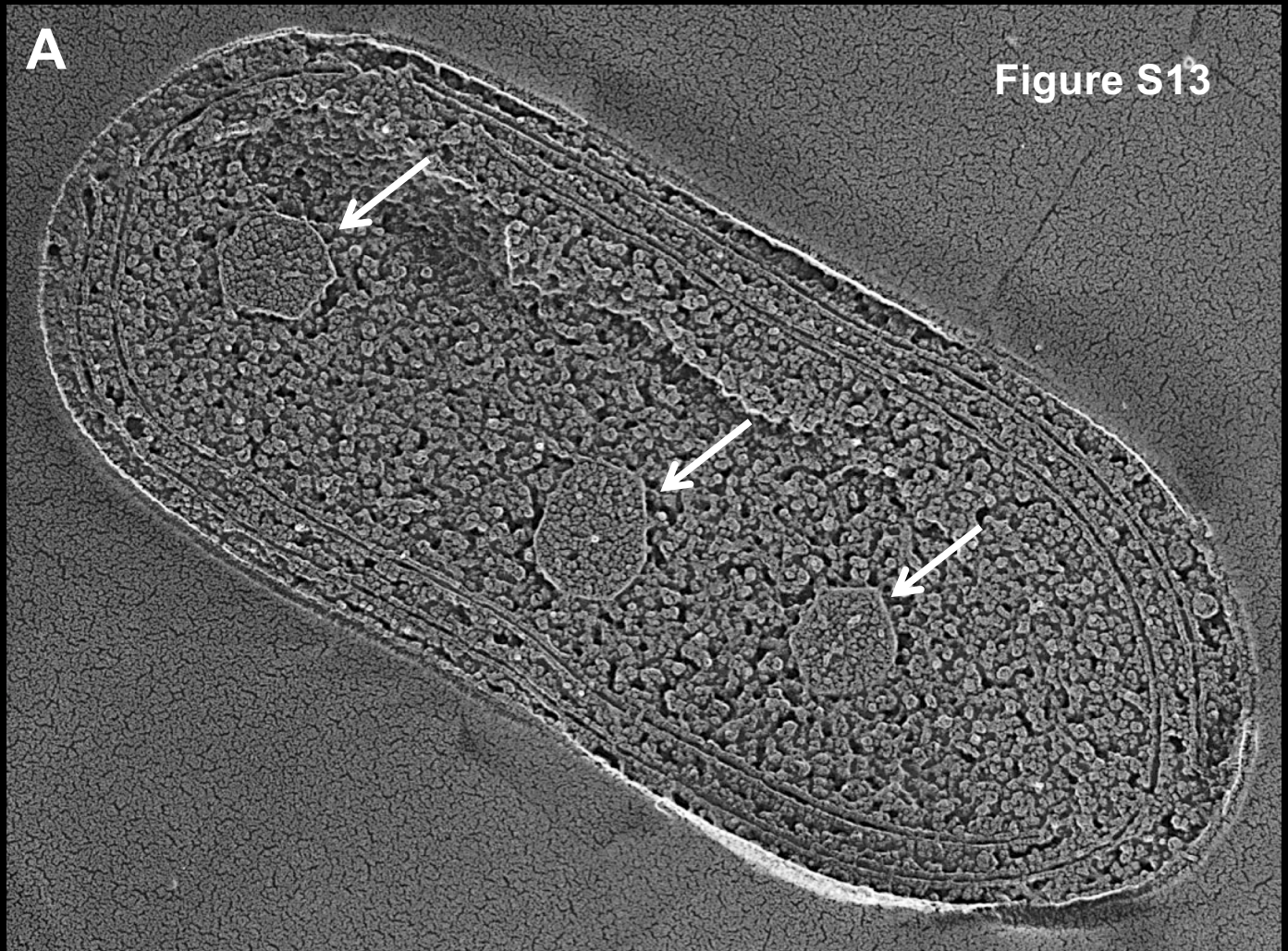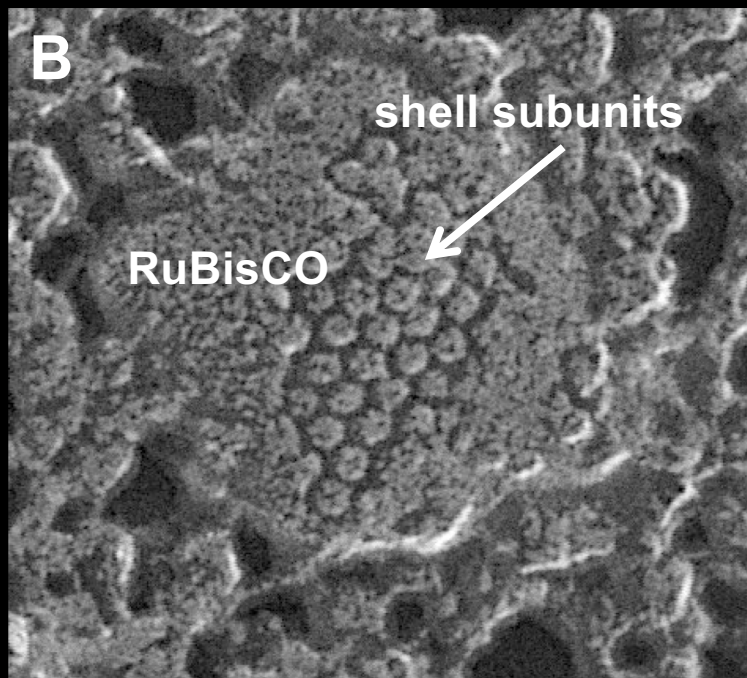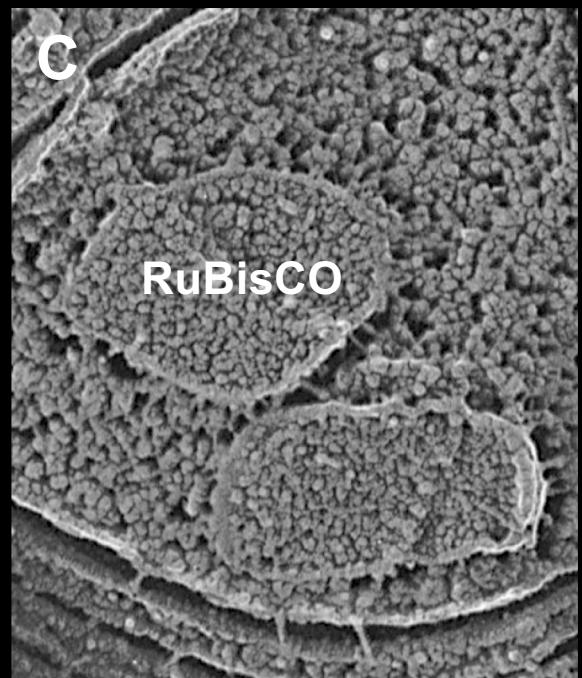

Supplement: dsz009_Supplementary_Data [file dsz009_supplementary_data.zip › dsz009-Suppl_data/Supplementary_Figure S13.pdf]
