# Supplementary material for: Analysis of an improved Cyanophora paradoxa genome assembly
Source: DNA Res. 2019 May 16;26(4):287–99. doi: 10.1093/dnares/dsz009 (PMC6704402; doi:10.1093/dnares/dsz009)
Supplement: dsz009_Supplementary_Data [file dsz009_supplementary_data.zip › dsz009-Suppl_data/Supplementary_Data.doc]

**Supplementary Data**

**Analysis of an improved *Cyanophora paradoxa* genome assembly**

Dana C. Price, Ursula W. Goodenough, Robyn Roth, Jae-Hyeok Lee, Thamali Kariyawasam, Marek Mutwil, Camilla Ferrari, Fabio Facchinelli, Steven G. Ball, Ugo Cenci, Cheong Xin Chan, Nicole E. Wagner, Hwan Su Yoon, Andreas P. M. Weber, and Debashish Bhattacharya

***RNA-Seq approach and results***

To quantify broad patterns of *C. paradoxa* gene expression in response to light, we performed a time-course differential gene-expression analysis using RNA-Seq data collected at six time points spanning a 16:8 light/dark cycle. The time points were 1hr after lights-on (ALO), 8hr ALO, 14hr ALO, 1hr after dark (AD), 5hr AD and 7hr AD. Genes with a significant change in expression (log_2_ fold-change ≥ 1.5) between any two time points were grouped according to KEGG pathway assignment, and expression plots were created to illustrate the overall response of all pathway members. Pathways with a clear upward or downward trend comprising the majority of assigned genes were retained for analysis.

Light exposure resulted in an overall up-regulation of genes shared between pathways involved in genetic information processing, including those responsible for DNA replication / damage repair (DNA replication, base excision repair, nucleotide excision repair, mismatch repair and homologous recombination) and the cell cycle progression (cell cycle and meiosis). In addition, we found degradation of amino acids (valine, leucine and isoleucine degradation, and lysine degradation) and biosynthesis of N- and O-linked glycans (N-glycan biosynthesis, various types of N-glycan biosynthesis, Other types of O-glycan biosynthesis) to be up-regulated along with constituents of the endocytosis and butanoate metabolism pathways (Fig. S7). In contrast, significant down-regulation was found for genes (Fig. S8) assigned to pathways involved in translation and protein trafficking (ribosome [ribosomal proteins], ribosome biogenesis in eukaryotes, aminoacyl-tRNA biosynthesis, protein export) along with isoprenoid biosynthesis (terpenoid backbone biosynthesis, carotenoid biosynthesis), cofactor metabolism (riboflavin metabolism, vitamin B6 metabolism, folate biosynthesis and porphyrin and chlorophyll metabolism), and amino acid metabolism (phenylalanine, tyrosine and tryptophan biosynthesis).

**Supplementary figures**

Figure S1. Maximum likelihood (ML) phylogeny of TOC75 and OEP80 that form the protein translocation channel in the outer membrane of the plastid envelope in Archaeplastida. This tree was generated using IQ-TREE with the best-fit LG + R7 model chosen under the Bayesian Information Criterion. Node labe­ls represent branch support determined by 1,000 rapid bootstraps (when >60%) as implemented in IQ-TREE. This tree demonstrates the ancient cyanobacterial origin of Toc75 in the Archaeplastida ancestor. Glaucophytes are in violet text, red algae in red text, green algae and plants in green text, cyanobacteria in blue text, and other taxa in black text. The tree was rooted on the branch leading to the Firmicutes and Proteobacteria.

Figure S2. *K*-mer Analysis of *Cyanophora paradoxa* Illumina data. *K*-mer analysis of Illumina reads from *C. paradoxa* using a variety of spectra under the expectations of a haploid or a diploid genome ([http://kmergenie.bx.psu.edu](http://kmergenie.bx.psu.edu/)). The predicted best fits are *k* = 107 for a haploid genome and *k* = 111 for a diploid genome. The red lines represent the fit of the complete statistical model of the histogram (erroneous *k*-mers + genomic *k*-mers). Under the diploid model, the blue line represents the heterozygous *k*-mers, whereas the green line represents the homozygous *k*-mers. The smaller peak at ca. ½ the *k*-mer coverage under the diploid model suggests that this glaucophyte is a diploid with low heterozygosity.

Figure S3. Repeat landscape of the *Cyanophora paradoxa* genome. Comparisons are made genomes from red algae (*Cyanidioschyzon merolae*, *Gracilariopsis chorda* and *Porphyra umbilicalis*) and green algae (*Micromonas commoda*, *Ostreococcus lucimarinus*, and *Chlamydomonas reinhardtii*). To minimize potential biases of misassembled repetitive regions, only genome assemblies with highly contiguous sequences or at chromosomal resolution are shown.

Figure S4. ML tree (built as in Fig. S1) of a 718-amino acid protein that encodes a putative bacterium-derived ABC transporter in *Cyanophora paradoxa*. The reverse frame encodes a 715-amino acid protein (NCBI top hit to *Oscillibacter* sp., *e*-val=2 x 10^-22^) annotated as a 30S ribosomal protein S5. Whether a spliced mRNA is transcribed and/or protein translated from each frame remains unknown.

Figure S5. ML tree (built as in Fig. S1) of GlgX. This gene encodes a putative glycogen debranching enzyme in *Cyanophora paradoxa* (and other Archaeplastida, and recipients of secondary/tertiary plastids) that is of chlamydial origin.

Figure S6. ML tree (built as in Fig. S1) of a eukaryote-derived gene (g22709b) in *Cyanophora paradoxa*. This gene encodes a complete eukaryotic glycogenin (GT8) domain involved in glycogen biosynthesis.­­

Figure S7. Genes in *Cyanophora paradoxa* that are up-regulated under light exposure. These gene are involved in various processes such as genetic information processing, including those responsible for DNA replication/damage repair (*DNA replication, base excision repair, nucleotide excision repair, mismatch repair* and *homologous recombination*) and the cell cycle progression (*cell cycle* and *meiosis*). See titles of each image for additional annotations.

Figure S8. Genes in *Cyanophora paradoxa* that are down-regulated under light exposure. These genes are involved in various processes such as protein trafficking (*ribosome* [ribosomal proteins], *ribosome biogenesis in eukaryotes*, *aminoacyl-tRNA biosynthesis*, *protein export)* along with isoprenoid biosynthesis (*terpenoid backbone biosynthesis*, *carotenoid biosynthesis*), cofactor metabolism (*riboflavin metabolism*, *vitamin B6 metabolism*, *folate biosynthesis*). See titles of each image for additional annotations.

Figure S9. RNA-Seq analysis of cell cycle genes in *Cyanophora paradoxa*. The expression patterns of well-studied cell cycle and other genes involved in cell proliferation are shown over the light (L) and dark (D) cycle at 1hr, 8hr, 14h under light and 1hr, 5hr, and 7hr under dark conditions­­.

Figure S10. Full eukaryotic tree that shows the position of *Cyanophora paradoxa* in the tree of life. This phylogeny was constructed using a partitioned 3,000 OG dataset from 115 publicly available eukaryote proteomes, as described in Price and Bhattacharya ^25^.

Figure S11. Alveolar cisternae in *Cyanophora paradoxa*. (A) P fracture faces (the bilayer leaflet contiguous to the cytoplasm or plasma membrane) of alveolar membranes, interconnected by sutures. pm, etched plasma membrane surface showing knobbly architecture. (B) E fracture faces (the bilayer leaflet contiguous to the lumen) of alveolar membranes, interconnected by sutures.

Figure S12. Quick-freeze deep-etch electron microscopy (QFDEEM) images of the *Cyanophora paradoxa* cell. (A) Cross-fractured alveolar cisternum showing its lumen (arrow). pm, etched surface of plasma membrane. (B) P fracture faces of alveolar membranes showing cisternal overlap (c.f. Kies ^59^).

Figure S13. Quick-freeze deep-etch electron microscopy (QFDEEM) images of carboxysomes in the cyanobacterium *Synechococcus* 7002. (A) Carboxysomes (arrows) delimited by shells. (B). Subunits of shell layer, each with a central pore, and RuBisCO. (C) RuBisCO surrounded by shells.

Figure S14. Quick-freeze deep-etch electron microscopy (QFDEEM) images of the *Cyanophora paradoxa* cell. The *C. paradaxa* plastid showing circular organization of thylakoids and central pyrenoid (pyr) surrounded by stroma.

Figure S15. Quick-freeze deep-etch electron microscopy (QFDEEM) images of the *Cyanophora paradoxa* cell. (A) Pyrenoid (pyr) of *C. paradaxa* with thylakoid termini in the stroma (arrows). (B) Pyrenoid of chlorophyte *Chlamydomonas reinhardtii* with thylakoids (T) traversing the matrix and either originating or terminating in a central disordered membranous domain.

Figure S16. Quick-freeze deep-etch electron microscopy (QFDEEM) images of the *Cyanophora paradoxa* and *Galdieria sulphuraria* cells. Phycobilisomes of *C. paradaxa* (A) and the larger phycobilisomes of the red alga *G. sulphuraria* (B). pyr, pyrenoid.

Figure S17. Quick-freeze deep-etch electron microscopy (QFDEEM) images of the *Cyanophora paradoxa* cell. Thylakoid fracture faces of *C. paradaxa* (A and C) and the cyanobacterium *Synechococcus* 7002 (B), showing rows of intramembranous particles (IMPs) on the E-faces (the leaflet contiguous to the thylakoid lumen) and a dense array of IMPs on the P-face (the leaflet contiguous to the stroma). In (A), the circular architecture of the thylakoid system is reflected in the four angles (1-4) of the IMP rows in successive fracture planes. phy, phycobilisomes.

Figure S18. Quick-freeze deep-etch electron microscopy (QFDEEM) images of the *Cyanophora paradoxa* cell. (A) *En face* view of 3 rows of phycobilisomes (arrow) showing their subunit organization, and adjacent IMP rows on a fractured E-face (left) of *C. paradoxa* thylakoids. (B) Mitochondrion of *C. paradoxa* with plate-like (flat) cristae.

Figure S19. Quick-freeze deep-etch electron microscopy (QFDEEM) images of the cyanobacterium *Synechococcus* 7002. (A) Onset of cleavage (arrows) with amorphous material in the indentation. (B) Later stage of cleavage showing fibrils (f) in the furrow.

Figure S20. Quick-freeze deep-etch electron microscopy (QFDEEM) images of the *Cyanophora paradoxa* cell. (A) The two *C. paradoxa* plastid envelope membranes between arrows. (B) Incipient furrow (arrow) in non-dividing cell, containing fibrils. (C) Cleavage furrow (arrow) in dividing cell, containing fibrils (f).

Figure S21. Detergent treatment of *Cyanophora paradoxa* cells. Control cell (A-D) and cell treated with 1% NP-40 non-ionic detergent (E-G). a and d, chlorophyll autofluorescence; B and F, SYBR green II (DNA-staining) signal, N: nucleus; c and g, DIC image; d, overlap of A and B, with yellow representing chloroplast DNA surrounding pyrenoid.

Figure S22. Quick-freeze deep-etch electron microscopy (QFDEEM) images of the *Cyanophora paradoxa* cell. Tangential fracture through cell anterior showing contractile vacuole (cv), mucocysts (m), Golgi (G), and alveoli (alv).

Figure S23. ML trees (built as in Fig. S1) of the peptidoglycan biosynthetic enzymes MraY and MurB in *Cyanophora paradoxa*. MraY is of cyanobacterial (presumably via EGT from the cyanobiont) origin, whereas MurB appears to be of non-cyanobacterial (prokaryotic) provenance.

Figure S24. ML trees (built as in Fig. S1) of phosphoenolpyruvate carboxykinase (PECK) and carbonic anhydrase (CA). These genes have non-cyanobacterial (prokaryotic) origins in Glaucophyta.

**Supplementary tables**

Table S1. List of 115 publicly available eukaryote proteomes that were used to generate the eukaryotic tree of life presented in this study.

Table S2. BLASTP output, *C. paradoxa* assembly v.2 queried against the v.1 proteome.

Table S3. BLASTP output, *C. paradoxa* assembly v.1 queried against the v.2 proteome.

Table S4. Proteins from *C. paradoxa* v.1 assembly lacking BLASTP hits to v.2 assembly (and thus removed from the proteome), with associated tBLASTN hits to assembled v.2 genome scaffolds and BLASTP top hits against the NCBI 'nr' database.

Table S5. Proteins from *C. paradoxa* v.2 assembly lacking BLASTP hits to v.1 assembly (and thus added to the proteome), with associated BLASTP top hits against the NCBI 'nr' database.

Table S6. The 4,049 (partially redundant) transcripts that encode bi-directional and partially or completely overlapping ORFs revealed by translation of the assembled *de novo* EST dataset from *Cyanophora paradoxa*. These genes were not identified during genome annotation because overlapping genes are not considered during gene prediction using Augustus.

Table S7. Results of phylogenomic analysis of peptidoglycan (PG) synthesis enzymes using the novel glaucophyte proteins models. Proteins of putative cyanobacterial (i.e., EGT) origin are shown in the blue field, whereas proteins of likely non-cyanobacterial provenance are in the red field. The IQ-TREE bootstrap support values for the affiliations are shown after the donor names. Proteins with an uncertain origin are in the white field at the bottom of the table. BLASTp output and *in silico* annotations are also shown.

Table S8. List of 34 well supported and 2 more weakly supported genes in *C. paradoxa* that are putatively of chlamydial origin. The gene model number, distribution in Archaeplastida, and *in silico* annotations are reported here. Chlamydial genes limited to *C. paradoxa* (with bootstrap support values for the relationship) are shown in the blue field, genes shared by *C. paradoxa* and Rhodophyta are in the red field, genes shared by *C. paradoxa* and Viridiplantae are in the green field, and genes shared by all three Archaeplastida are in the brown field.
